# The Epidemiology and Geographic Distribution of Relapsing Fever Borreliosis in West and North Africa, with a Review of the *Ornithodoros erraticus* Complex (Acari: Ixodida)

**DOI:** 10.1371/journal.pone.0078473

**Published:** 2013-11-04

**Authors:** Jean-François Trape, Georges Diatta, Céline Arnathau, Idir Bitam, M’hammed Sarih, Driss Belghyti, Ali Bouattour, Eric Elguero, Laurence Vial, Youssouph Mané, Cellou Baldé, Franck Pugnolle, Gilles Chauvancy, Gil Mahé, Laurent Granjon, Jean-Marc Duplantier, Patrick Durand, François Renaud

**Affiliations:** 1 Institut de recherche pour le développement, Laboratoire de Paludologie Et Zoologie Médicale, Dakar, Senegal; 2 Institut de Recherche pour le Développement, UMR (CNRS IRD) MIVEGEC, Montpellier, France; 3 Institut Pasteur d’Algérie, Laboratoire d’Écologie des Systèmes Vectoriels, Algiers, Algeria; 4 Institut Pasteur du Maroc, Laboratoire des Maladies Vectorielles, Casablanca, Morocco; 5 Université Ibn Tofail, Département de Biologie, Faculté des Sciences, Kénitra, Morocco; 6 Institut Pasteur de Tunis, Service d’Entomologie Médicale, Tunis, Tunisia; 7 Institut Pasteur de Guinée, Laboratoire d’Entomologie Médicale et de Vénimologie, Kindia, Guinea; 8 Institut de Recherche pour le Développement, UMR Hydrosciences, Montpellier, France; 9 Institut de Recherche pour le Développement, Centre de Biologie et de Gestion des Populations, Dakar, Senegal; Umeå University, Sweden

## Abstract

**Background:**

Relapsing fever is the most frequent bacterial disease in Africa. Four main vector / pathogen complexes are classically recognized, with the louse *Pediculus humanus* acting as vector for *B. recurrentis* and the soft ticks *Ornithodoros sonrai*, *O. erraticus* and *O. moubata* acting as vectors for *Borrelia crocidurae, B. hispanica* and *B. duttonii*, respectively. Our aim was to investigate the epidemiology of the disease in West, North and Central Africa.

**Methods And Findings:**

From 2002 to 2012, we conducted field surveys in 17 African countries and in Spain. We investigated the occurrence of *Ornithodoros* ticks in rodent burrows in 282 study sites. We collected 1,629 small mammals that may act as reservoir for *Borrelia* infections. Using molecular methods we studied genetic diversity among *Ornithodoros* ticks and *Borrelia* infections in ticks and small mammals. Of 9,870 burrows investigated, 1,196 (12.1%) were inhabited by *Ornithodoros* ticks. In West Africa, the southern and eastern limits of the vectors and *Borrelia* infections in ticks and small mammals were 13°N and 01°E, respectively. Molecular studies revealed the occurrence of nine different *Ornithodoros* species, including five species new for science, with six of them harboring *Borrelia* infections. Only *B. crocidurae* was found in West Africa and three *Borrelia* species were identified in North Africa: *B. crocidurae*, *B. hispanica*, and *B. merionesi*.

**Conclusions:**

*Borrelia* Spirochetes responsible for relapsing fever in humans are highly prevalent both in *Ornithodoros* ticks and small mammals in North and West Africa but *Ornithodoros* ticks seem absent south of 13°N and small mammals are not infected in these regions. The number of *Ornithodoros* species acting as vector of relapsing fever is much higher than previously known.

## Introduction

Relapsing fever has long been recognized as major cause of disease and death in several regions of Africa [1-5]. Initially discovered in India in 1907 [6], the responsibility of *Pediculus humanus* in the transmission of *Borrelia recurrentis*, the Obermeier’s spirochete, was confirmed the following year in Algeria [7]. Dramatic epidemics of louse-borne relapsing fever (LBRF) responsible for several millions of cases and a high fatality rate occurred throughout Africa after World Wars I and II when French and British colonial soldiers infected in Europe or North Africa returned to their countries [8-12]. More recently, several epidemics occurred in Sudan and LBRF still persists in the mountains of Ethiopia where it is endemic and can account for up to 27% of hospital admissions [5,13-16]. Tick-borne relapsing fever (TBRF) was first recognized in East Africa in 1904 [17,18], in North Africa in 1928 [19], and in West Africa in 1932 [20]. Studies conducted between 1905 and 1960 progressively established the classical picture of TBRF in Africa, with three different vector/pathogen complexes involving soft ticks (*Argasidae*) of the genus *Ornithodoros*. In the savanna areas of eastern and southern Africa, from 16°N (Erythrea) to 34°S (Cape province, South Africa), TBRF is caused by *Borrelia duttonii* with *O. moubata* s.l. as vector [21]. In the wild, the two vector species of the *O. moubata* complex (*O. moubata s.s.* and *O. porcinus*) lives in large animal burrows of antbears, warthogs and porcupines [22]. They have adapted secondarily to human dwellings and domestic animal shelters, where they live in the cracks of walls and floors. There is no known mammal reservoir of the disease and *O. moubata* s.l. act both as the vector and the only reservoir of *B. duttonii* [23]. The annual incidence of the disease may reach up to 384 per thousand among children <1 year of age and 163 per thousand in children <5 years of age in Tanzania [24], and a lethality rate of 16% among pregnant women suffering from the disease has been reported from Rwanda [25]. In North Africa, from Morocco to Algeria and Tunisia, TBRF is classically caused by *Borrelia hispanica* with *O. erraticus* as vector and small mammals as reservoir host [2,26-31]. *O. erraticus* is found both in large and small burrows, under stones, and has adapted to domestic animal shelters. Most human infections occur during summer among people sleeping in the fields or in farm buildings [32]. In West Africa, TBRF is caused by *Borrelia crocidurae*, with *O. sonrai* as vector and rodents and insectivores as reservoir host [33-37]. Most reports of the disease are from Senegal, but the few available data on the occurrence of the vector also include Mali, Mauritania, southern Morocco, Niger, Chad, Egypt and Kenya [23]. *O. sonrai* inhabits rodent burrows and like the other *Ornithodoros* species, it is a rapid feeder, blood meals lasting only a few minutes. People are generally infected during their sleep, when burrows open into their bedrooms [36].

The high incidence of TBRF in places where it was specifically investigated contrasts with the rarity of reports from other areas, suggesting than most cases remain undiagnosed because usually confused with malaria whose diagnosis is usually based only on clinical symptoms [38]. In Senegal, as a result of the ongoing drought since the 1970s, the tick has colonized the Sudan savanna, with the 750 mm isohyet as southern limit [36]. Rodent burrows colonized by *O. sonrai* occur in 87% of villages north of 13°30’N. In these areas, we have shown that the average incidence of TBRF at the community level is the highest described for any bacterial disease, reaching 11 per 100 person-years [39]. In out-patients clinics, TBRF is the second most common cause of fever after malaria [38]. These observations in Senegal have prompted us to investigate the distribution and epidemiology of TBRF in West, North, and Central Africa. Here we present the result of studies conducted between 2002 and 2012 in 17 African countries.

## Methods

### Ticks sampling

To investigate the geographic distribution of *Ornithodoros* ticks, we conducted four series of studies

i) One transect study along the 14th parallel in Senegal, Mali, Burkina Faso, Niger and Chad, from 16°W (near the Atlantic coast of Senegal) to 22°E (near the border of Sudan in Chad). Sampling was conducted at each two degrees of longitude (i.e. at 16°W/14°N, 14°W/14°N, 12°W/14°N, and up to 22°E/14°N), either at the exact meridian/parallel junction point as determined with a Global Positioning System (GPS) receiver, or depending on the accessibility of this point and local environment, within a 10’ radius around it (i.e. maximum distance: 20 km). The 14th parallel was selected since it was entirely located within the limits of the supposed range of O. sonrai (presumed southern limit: 750 mm isohyet, period 1970-2002) [36].
ii) Three North/South transect studies along three meridians: 12°W (in Morocco, Mauritania, Senegal, and Guinea), 2°E (in Mali, Niger, Burkina Faso and Benin), and 14°E (in Chad and Cameroon). Sampling was undertaken at each degree of latitude, from 10°N to 28°N, 6°N to 19°N, and 8°N to 14°N, respectively. In Morocco, the transect was further continued SW/NE up to the Mediterranean coast.
iii) Based on the results of the transect studies, additional surveys were conducted in Senegal, The Gambia, Mauritania, Mali and Niger in order to determine either on a 10 km square scale (Western Senegal) or on a half or quarter square degree scale the southern and eastern limits of the geographic distribution of Ornithodoros species.
iv) We also undertook additional surveys in selected areas of Morocco, Algeria, Tunisia, Niger, Guinea, Guinea Bissau, Liberia, Ivory Coast, Burkina Faso and Togo. All surveys were conducted in predefined sites based on their geographic position. One site south of Spain, where the first studies on TBRF in Europe were conducted in the 1920s by Sadi de Buen [32], was also sampled for comparison of local tick population with African Ornithodoros species.

For each sampling site, as a general rule 30 to 60 burrows were investigated (60 burrows during transect studies when all burrows were negative for the presence of *Ornithodoros* ticks, 30 burrows when one or more burrows were positive; only 30 burrows even when all were negative during additional surveys), except in sites rapidly positive during additional surveys in North Africa where only 5 to 15 burrows were investigated. Ticks were collected by introducing a flexible tube inside burrows and aspirating their contents using a portable petrol-powered aspirator. After collection, they were immediately stored in absolute ethanol (one tube per positive burrow) until morphological determination and DNA extraction. As a general rule, two or three sampling stations were selected in each site, including one village (except in most Saharan and North African sites) where several houses were surveyed for the occurrence of ticks both inside burrows and in cracks in the floor or walls. Outside villages, sampling stations were selected according to local environment either in natural or human-impacted ecosystems and croplands or both of them.

### Small mammals sampling

To investigate the reservoir of *Borrelia*, we captured rodents and insectivores in Senegal, Mali, Benin, Niger, Chad, Cameroon, Mauritania and Morocco. These collections were made along 12°W, 2°E and 14°E meridians and in further sites in Senegal, Mali and Morocco. Animals were trapped alive with lattice-work traps baited with peanut butter or onions. We adopted the method of trap lines for captures, with a 10-meter space between traps. In order to collect both diurnal and nocturnal species traps were placed in the afternoon and withdrawn late in the morning. Approximately 200 traps/days of captures were made in each site. In addition, hand captures of rodents were performed by night in most sites and during travel between sites. We necropsied trapped animals in the field by means of cervical dislocation and drew 1mL of blood from each by cardiac puncture. We immediately prepared a thick blood film for detection of *Borrelia*. Samples of brain were stored in nitrogen then at -80°C for further intraperitoneal inoculation to white mouse as previously described [40] and/or *Borrelia* molecular studies as described below.

 The nomenclature here adopted follows Wilson & Reeder [41] unless otherwise mentioned [42]. Classical body measurements were taken and some individual rodents were kept alive for further chromosomal analyses, as described in Granjon & Dobigny [43]. Most of the rodents of the Sahelo-Sudanian region could be diagnosed to the species level based on our morphological, ecological and biogeographical knowledge on rodents from this geographic area [42]. However, molecular or chromosomal data were necessary to confirm unambiguously the specific determination of some specimens of the genera *Arvicanthis*, *Gerbillus*, *Gerbilliscus*, *Mastomys*, and *Taterillus* especially. This was done according to methods previously described [43-47]. Shrews were determined on the basis of external and skull criteria [Granjon et al, unpublished]. A selection of voucher specimens was kept in formalin or ethanol and is housed at IRD (UMR 22, Dakar and Montpellier). Organ samples as well as DNA and bone marrow cells extracts that have been used for molecular/cytogenetic studies are also kept in the IRD tissue collection. 

### DNA isolation and PCR amplification in ticks

Ticks were individually washed in three sterile water baths, air dried and collected in sterile microtubes. DNA was individually crushed by shaking with a bead beater (mixer mill MM301, Qiagen, Hilden, Germany), and then DNA was isolated and purified using the DNeasy Blood and Tissue extraction Kit (Qiagen). 

 For each tick, 16S rRNA was amplified by PCR with Tm16S+1 (5’-CTGCTCAATGATTTTTTAAATTGC-3’) and Tm16S-1 (5’-CCGGTCTGAACTCAGATCATGTA-3’) primers designed by Fukunaga et al. [48]. The sequenced product size was around 450 bp long and PCRs were performed with the Multiplex PCR Kit (Qiagen) in a 25 µl volume containing 12.5 µl of 2x Multiplex PCR Master Mix (Qiagen), 2 µM of each Primer, 2.5 µl of Q-solution and 4 µl (40-100 ng/µL) of DNA template. The amplification cycle involved a denaturation step at 95°C for 15 min followed by 10 cycles of 1 min at 92°C, 1.5 min at 48°C, and 1.5 min at 72°C and 32 cycles of 1 min at 92°C, 1.5 min of 54°C, and 1.5 min of 72°C. A final extension step was carried out for 10 min at 72°C. The amplified products were detected by electrophoresis on 1.5% agarose gel in TAE 0.5X buffer and staining with Envision (Amaresco). Remaining reaction mixtures were stored at -20°C for direct sequencing. 

### Detection of *Borrelia* infections in ticks and small mammals

DNA isolation was conducted as above. *Borrelia* detection was based on nested PCR amplification of a 350 bp fragment of the flagella gene (FLA). This gene encodes the periplasmic protein peculiar to *Borrelia*. The amplification was performed using primers (Bfpad and Bfpdu for the first PCR, Bfpbu and Bfpcr for the second PCR) designed for *B. duttonii* [48]. Each PCR was performed in a 25 µl volume containing 5µl 5X buffer (Promega), 2 mM MgCl_2_, 200 µM of each dNTP, 0.2 µM of each primer and 2.5 unit of GoTaq DNA polymerase (Promega). 3µl of DNA template was added in the first reaction and 1 µl of the first amplified product was added in the second reaction. Amplification cycles consisted of an initial DNA denaturation step at 94°C for 3 min followed by 30 cycles of 40 sec at 94°C, 40 sec at 55°C for the first PCR and 51°C for the second PCR, and 40 sec at 72°C. A final extension step was carried out for 10 min at 72°C. 

 For the 16S-23S ribosomal RNA intergenic spacer (IGS), *rrs rrlA* intergenic spacer IGS/F and IGS/R for the first PCR and *rrs rrlA* IGS/Fn and IGS/Rn for the second PCR were amplified as previously described [49]. Each PCR was performed in a 25 µl volume containing 5µl 5X buffer (Promega), 2 mM MgCl_2_, 200 µM of each dNTP, 5 picomoles of each primer and 2.5 unit of GoTaq DNA polymerase (Promega). 2µl of DNA template was added in the first reaction and 1 µl of the first amplified product was added in the second reaction. Amplification cycles consisted of an initial DNA denaturation step at 94°C for 3 min followed by 35 cycles of 30 sec at 94°C, 30 sec at 56°C, 30 sec at 72°C and a final extension 5 min at 72°C for the first PCR. For the second PCR, amplification cycles consisted of an initial DNA denaturation step at 94°C for 3 min followed by 40 cycles of 30 sec at 94°C, 30 sec at 60°C, 30 sec at 72°C and a final extension 5 min at 72°C for the first PCR. A final extension step was carried out for 5 min at 72°C. The amplified products were detected by electrophoresis as above. Negative control was included in each nested PCR analysis to monitor contamination and false-positive amplification. To rule out amplicon carry-over, nucleotide-free water negative control was used throughout the steps of the protocol. The sequence-derived data reported herein were authentified as negative controls introduced in every PCR experiment remained negative, excluding the possibility of cross-contamination during the experiments. The amplified products for 16S rDNA, IGS and FLA were directly sequenced by Eurofins (Ebersberg, Germany).

### Sequence alignment and phylogenetic inferences

All sequences obtained were aligned using ClustalW (v.1.8.1 in BioEdit v.7.0.5.3.) [50]. Maximum Likelihood (PhyML) tree reconstruction was conducted from the 16S rRNA aligned sequences (457 nucleotides) of ticks. For each *Borrelia* of soft tick, the FlaB gene (FLA, 269 nucleotides) and Intergenic Spacer (IGS, 510 nucleotides) sequences were concatenated for the phylogenetic analyses (PhyML). For each dataset, we used Modeltest 3.4 [51] to select the appropriate model of molecular evolution. The best-fitting ML model was HKY85 [52] for 16S rRNA sequences and GTR (General Time Reversible + Gamma distribution) for IGS-FLA concatened sequences. The highest-likelihood DNA and corresponding bootstrap support values were obtained by PhyML (freely available at http://mobyle.pasteur.fr/cgi-bin/portal.py) using NNI (Nearest Neighbor Interchange) branch swapping and 100 bootstrap replicates [53].

Molecular distances with standard error estimate were conducted using the Kimura 2-parameter model among *Ornithodoros* 16S sequences and among *Borrelia* concatened IGS-FLA sequences as used in other phylogenetical pathogens/vectors studies [54,55]. In the final dataset, the analysis involved 165 different sequences grouping 821 16s RNA tick sequences and 105 different sequences grouping 216 IGS-FLA *Borrelia* sequences. Evolutionary analyses were conducted in MEGA 5 [56] and all positions containing gaps and missing data were eliminated.

 Phylogenetic analyses of *Ornithodoros moubata* 16S rRNA (GenBank accession number AB073679), *O. porcinus* (GenBank acc. no. AB105451), *O. turicata* (GenBank acc. no. L34327), and *O. parkeri* (GenBank acc. no. EU009925) were treated as outgroups. *Borrelia crocidurae* (GenBank acc. no. GU350723 and NC017808), *B. duttonii* (GenBank acc. no. DQ000279 and DQ346833)*, B. hispanica* (GenBank acc. no. GU350718 and GU357614), *B. merionesi* (GenBank acc. no. JX257047 and JX257050) and *B. recurrentis* (GenBank acc. no. DQ000277 and DQ346814) for the IGS and FLA sequences were concatened for the phylogenetic analyses of spirochetes.

 In the goal of molecular diagnostic of *Ornithodoros* species of North and West African countries, we determined Single Nucleotide Polymorphism (SNP) based on 16S rRNA sequences using parsimony-informative site.

### Analysis of environmental factors

 We investigated relationship between environmental factors (latitude, longitude, mean annual temperature, mean annual rainfall [57], elevation and distance to the seashore) and tick species distribution using a linear discriminant analysis. All calculations were performed with R software [58].

### Nomenclatural acts

 The electronic edition of this article conforms to the requirements of the amended International Code of Zoological Nomenclature, and hence the new names contained herein are available under that Code from the electronic edition of this article. The published work and the nomenclatural acts it contains have been registered in ZooBank, the online registration system for the ICZN. The ZooBank LSIDs (Life Science Identifiers) can be resolved and the associated information viewed through any standard web browser by appending the LSID to the prefix http://zoobank.org/. The LSID for this publication is: urn:lsid:zoobank.org:pub:583BB2C2-B859-4EB5-85D7-70945C923642. The electronic edition of this work was published in a journal with an ISSN, and has been archived and is available from the following digital repositories: PubMed Central, LOCKSS.

 The holotypes and paratypes of the new species are deposited at the Institut Royal des Sciences Naturelles de Belgique, Brussels, Belgium (IRSNB). Sequences obtained from a segment of leg of one or several specimens of the type series are deposited in GenBank.

### Ethics Statement

 The study protocol was approved by the Steering Committee of the IRD Special Programme Evolution Climatique et Santé (IRD, Montpellier, France), reference project ATI-ECS-07-H/2002. Animals were treated in a humane manner, and in accordance with authorizations and guidelines of the American Society of Mammalogists (Animal Care and Use Committee 1998). Captures of rodents and insectivores (Senegal, Mali, Benin, Niger, Cameroon, Chad, Mauritania, Morocco) and ticks (Senegal, Mali, Burkina Faso, Niger, Cameroon, Chad, Guinea Bissau, Guinea, Gambia, Liberia, Côte d’Ivoire, Togo, Benin, Mauritania, Morocco, Algeria, Tunisia, Spain) excluded national parks and protected areas, did not involve endangered or protected species (CITES, UICN, and national guidelines), and were conducted as part of research agreements between IRD, national institutions, national ministries of health and/or scientific research. All tick, rodent and insectivores captures inside houses and private lands were conducted after the owner of the house and land gave permission to conduct the study on this site.

## Results

### Geographic distribution of *Ornithodoros* ticks

We investigated the occurrence of *Ornithodoros* ticks in 9,870 burrows from 484 sampling stations in 282 study sites in 12 West African countries (Mauritania, Senegal, The Gambia, Guinea Bissau, Guinea, Mali, Burkina Faso, Niger, Ivory Coast, Benin, Togo, Liberia), three North African countries (Morocco, Algeria, Tunisia), two Central African countries (Cameroon, Chad), and one European country (Spain) (Table S1). 

**Figure 1:**
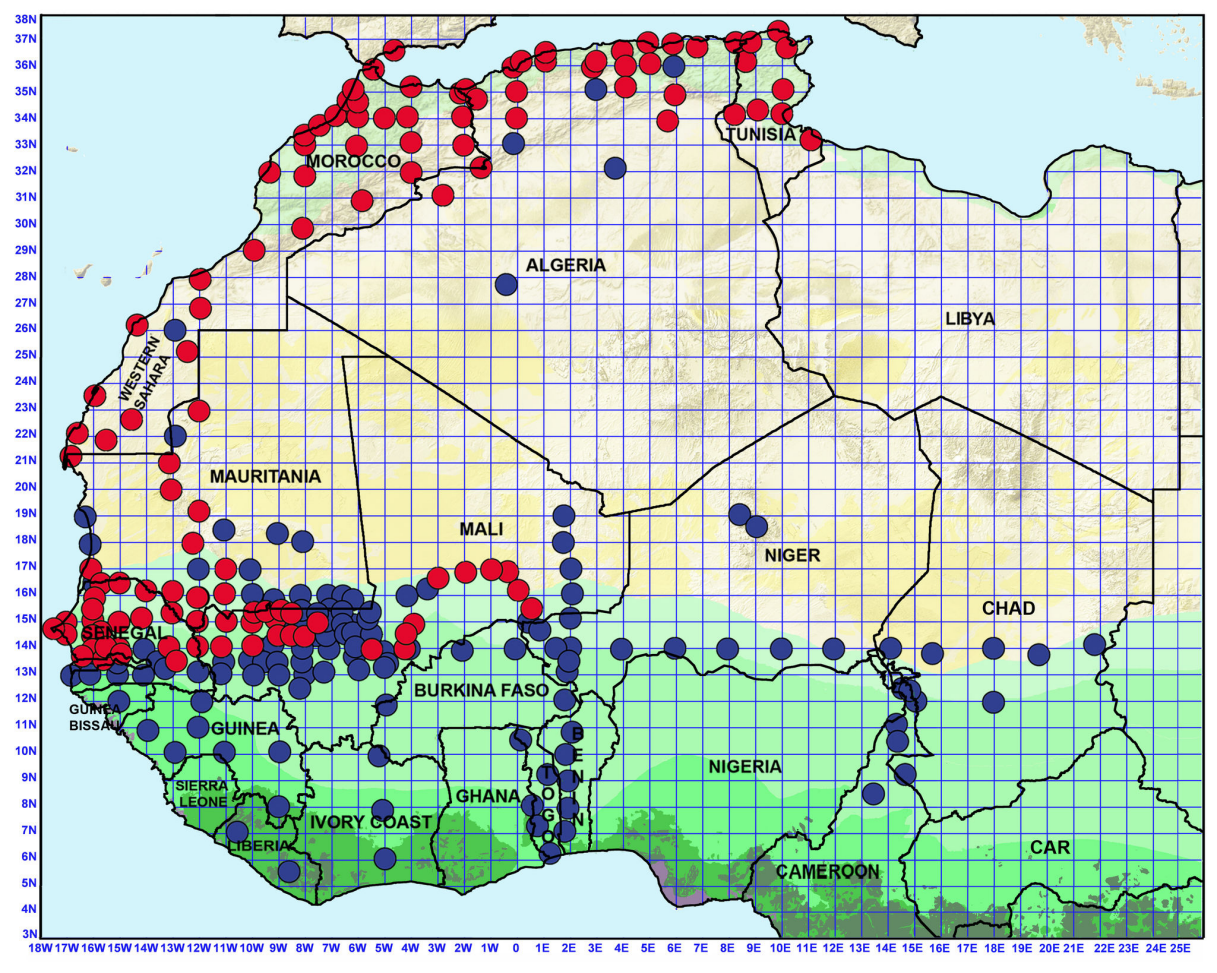
Map of northwestern Africa with the location of study sites positive (red circles) or negative (blue circles) for the occurrence of *Ornithodoros* ticks in small mammal burrows. At least 30 burrows were investigated in each negative site.

*Ornithodoros* ticks morphologically attributable to *O. sonrai*, *O. erraticus* or *O. normandi* were collected in 136 sites distributed in Morocco, Algeria, Tunisia, Mauritania, Senegal, The Gambia, Mali and Spain (Table 1). In West and Central Africa, all burrows located east of 01°E and south of 13°N were negative (Figure 1). The southernmost limits were 13°32’N, 13°35’N and 13°54’N in Senegal, The Gambia and Mali, respectively, and the easternmost limit south of Sahara was 00°34’E in Mali. In all countries where we found *Ornithodoros* ticks, their distribution was generally contiguous, with rarely a negative site between two positive sites, and thus it has been possible to establish in the field precise distribution limits (i.e. at a few km scale in western Senegal and at one quarter degree scale in other areas of Senegal and in western Mali). In central and eastern Mali, we found *Ornithodoros* ticks only in burrows located close to the flood plain of the Niger River. In other areas, the occurrence of *Ornithodoros* ticks in burrows was independent of any hydrographic system either active or fossil. Based on classical morphological criteria, all *Ornithodoros* ticks collected in burrows were attributable either to *O. sonrai* (Senegal, The Gambia, Mali, Mauritania, and the most arid areas of Morocco, Algeria and Tunisia), *O. erraticus* (northern Morocco, northwestern Algeria and a few ticks from northern Tunisia) or *O. normandi* (northeastern Algeria and northern Tunisia). We never observed *O. moubata*.

**Table 1:** Number of sites and burrows investigated and number and proportion positive for the presence of *Ornithodoros* ticks.

Country	No. of burrows			No. of sites	
	Investigated	Positive (%)		Investigated	Positive (%)
Algeria	435	74 (17.0)		24	19 (79.2)
Morocco	605	239 (39.5)		34	33 (97.1)
Tunisia	82	23 (28.1)		9	9 (100)
Mauritania	905	96 (10.6)		30	12 (40.0)
Senegal	2.861	474 (16.6)		61	37 (60.7)
The Gambia	30	15 (50.0)		2	2 (100.0)
Mali	2.458	273 (11.1)		69	23 (33.3)
Burkina Faso	210	0 (0.0)		4	0 (0.0)
Niger	631	0 (0.0)		13	0 (0.0)
Benin	300	0 (0.0)		5	0 (0.0)
Togo	150	0 (0.0)		5	0 (0.0)
Ivory Coast	90	0 (0.0)		3	0 (0.0)
Guinea	270	0 (0.0)		7	0 (0.0)
Guinea Bissau	30	0 (0.0)		1	0 (0.0)
Liberia	60	0 (0.0)		2	0 (0.0)
Chad	458	0 (0.0)		8	0 (0.0)
Cameroon	278	0 (0.0)		4	0 (0.0)
Spain	17	2 (11.7)		1	1 (100.0)
Total	9.87	1,196 (12.1)		282	136 (48.2)

### *Borrelia* infections in small mammals

A total of 1,629 small mammals belonging to 8 families (rodents: Ctenodactylidae, Dipodidae, Muridae, Nesomyidae, Sciuridae; insectivores: Erinaceidae, Soricidae; carnivores: Mustelidae) were collected in Senegal, Mauritania, Mali, Niger, Benin, Chad, Cameroon and Morocco (Table S2). As shown in Table 2, these specimens belonged to 50 identified species and a few additional undetermined species. A thick blood film was prepared in 1,270 specimens (Senegal: 80; Mauritania: 224; Morocco: 134; Mali: 413; Niger: 95; Benin: 101; Chad: 52; Cameroon: 171) and brain samples of 1,089 specimens (Senegal: 92; Mauritania: 176; Mali: 322; Niger: 115; Benin: 152; Chad: 52; Cameroon: 180) were inoculated to white mice. As previously reported, brain samples of the 140 specimens collected in Morocco were also studied by PCR [40]. A *Borrelia* infection was demonstrated in 57 animals from Senegal, Mali, Mauritania or Morocco (4.1 % of the 1,386 specimens tested by at least one method) belonging to 13 rodent species and two insectivore species (Table 2). All infected rodents and insectivores were collected in sites or areas where we documented the presence of *Ornithodoros* ticks. In these areas, we tested 868 animals and the proportion of those found infected by thick blood film examination, brain inoculation and PCR was 3.3% (28/850), 4.2% (21/499), and 8.6% (12/140), respectively. Except for Senegal, where 22.8% (21/92) of brain inoculations to white mice were positive (all were performed within a few days after sampling), all other brain inoculations to white mice were negative, including those from specimens from Mali and Mauritania with positive thick blood films, this certainly in relation to badly preserved spirochetes in brain tissue after sampling. 

**Table 2:** List of small mammals collected and number of specimens found infected by *Borrelia*.

Family	Species	No. collected	No. Tested	No. Infected *
**Rodents**				
Ctenodactylidae	*Felovia vae*	1	1	0
Dipodidae	*Jaculus jaculus*	37	36	0
Muridae	*Acomys airensis*	38	28	0
	*Acomys chudeaui*	17	14	0
	*Arvicanthis ansorgei*	3	3	0
	*Arvicanthis niloticus*	26	26	1 (I:1)
	*Arvicanthis rufinus*	5	0	0
	*Arvicanthis* spp.	51	38	0
	*Desmodilliscus braueri*	11	8	0
	*Gerbilliscus gambianus*	5	4	0
	*Gerbilliscus kempi*	4	0	0
	*Gerbilliscus* spp.	54	45	0
	*Gerbillus campestris*	59	59	4 (B:2, P:3)
	*Gerbillus gerbillus*	42	37	1 (B:1)
	*Gerbillus henleyi*	1	1	0
	*Gerbillus hoogstraali*	12	12	1 (B:1, P:1)
	*Gerbillus pyramidum*	28	28	0
	*Gerbillus nanus*	25	21	0
	*Gerbillus nigeriae*	28	26	0
	*Gerbillus occiduus*	29	29	1 (P:1)
	*Gerbillus tarabuli*	107	90	2 (B:2)
	*Gerbillus* spp.	41	20	0
	*Lemniscomys barbarus*	1	1	0
	*Lemniscomys bellieri*	2	0	0
	*Lemniscomys zebra*	10	8	0
	*Lemniscomys* spp.	14	14	0
	*Mastomys erythroleucus*	*205*	179	9 (B:2, I:8)
	*Mastomys huberti*	76	76	1 (B:1)
	*Mastomys kollmannspergeri*	*38*	38	0
	*Mastomys natalensis*	220	164	7 (B:7)
	*Mastomys* spp.	26	18	0
	*Meriones libycus*	1	1	0
	*Meriones shawi*	14	14	6 (B:1, P:6)
	*Mus musculus*	23	15	1 (I:1)
	*Pachyuromys duprasi*	1	0	0
	*Praomys daltoni*	180	175	11 (B:11)
	*Praomys derooi*	15	12	0
	*Rattus norvegicus*	2	2	0
	*Rattus rattus*	18	15	2 (I:2)
	*Taterillus arenarius*	7	5	0
	*Taterillus congicus*	2	2	0
	*Taterillus gracilis*	8	6	0
	*Taterillus lacustris*	7	5	0
	*Taterillus petteri*	2	2	0
	*Taterillus* spp.	13	10	0
	*Uranomys ruddi*	15	14	0
Nesomyidae	*Cricetomys gambianus*	7	4	0
Sciuridae	*Atlantoxerus getulus*	3	3	0
	*Heliosciurus gambianus*	*1*	1	0
	*Xerus erythropus*	6	3	0
**Insectivores**				
Erinaceidae	*Atelerix albiventris*	8	2	0
	*Atelerix algirus*	6	6	1 (P:1)
	*Hemiechinus aethiopicus*	*3*	2	0
Soricidae	*Crocidura olivieri*	41	33	9 (I:9)
	*Crocidura viaria*	9	9	0
	*Crocidura* spp.	20	20	0
**Carnivores**				
Mustelidae	*Ictonyx striatus*	1	1	0

* B: Thick blood film examination; I: white mice inoculation with brain tissue; P: PCR with brain tissue (Morocco specimens only).

### Molecular analysis of *Ornithodoros* ticks and *Borrelia* infections

A total of 1,801 *Ornithodoros* ticks from 113 study sites distributed in 7 African countries (Mali, Senegal, The Gambia, Mauritania, Morocco, Algeria and Tunisia) and in Spain were tested for molecular species determination and associated *Borrelia* infection. We observed the presence of *Borrelia* infections in 295 ticks (16.4%) distributed in all countries and 37.2% of sites (42/113). We obtained 820 sequences of 16S rRNA of *Ornithodoros* ticks, 216 concatened partial sequences of FlaB gene, and partial sequences of Intergenic spacer for the *Borrelia* detected (Table 3). 

**Table 3:** Distribution and prevalence of infected *Ornithodoros* ticks by *Borrelia* in seven North and West Africa countries and in one locality of Spain.

Country	Sites	No. of ticks tested	No. of infected ticks	Prevalence	No. of IGS-FLA *Borrelia* concatened sequences (786 nu.)	No. of 16S rDNA tick sequences (457 nu.)
Algeria	18	199	5	2.5	2	183
Gambia	1	8	4	50.0	4	1
Mali	18	273	34	12.5	26	88
Mauritania	9	72	7	9.7	7	21
Morocco	29	415	40	9.6	24	188
Senegal	28	768	199	25.9	146	281
Spain	1	4	2	50.0	2	1
Tunisia	9	62	4	6.5	4	57
Total	113	1801	295	16.4	215	820

The number of partial sequences obtained of concatened Intergenic spacer – FlaB gene (IGS-FLA) for *Borrelia* and 16S rDNA for ticks are shown. No.: number; Nu: nucleotide.

### Phylogenetic analysis of *Ornithodoros* ticks

**Figure 2:**
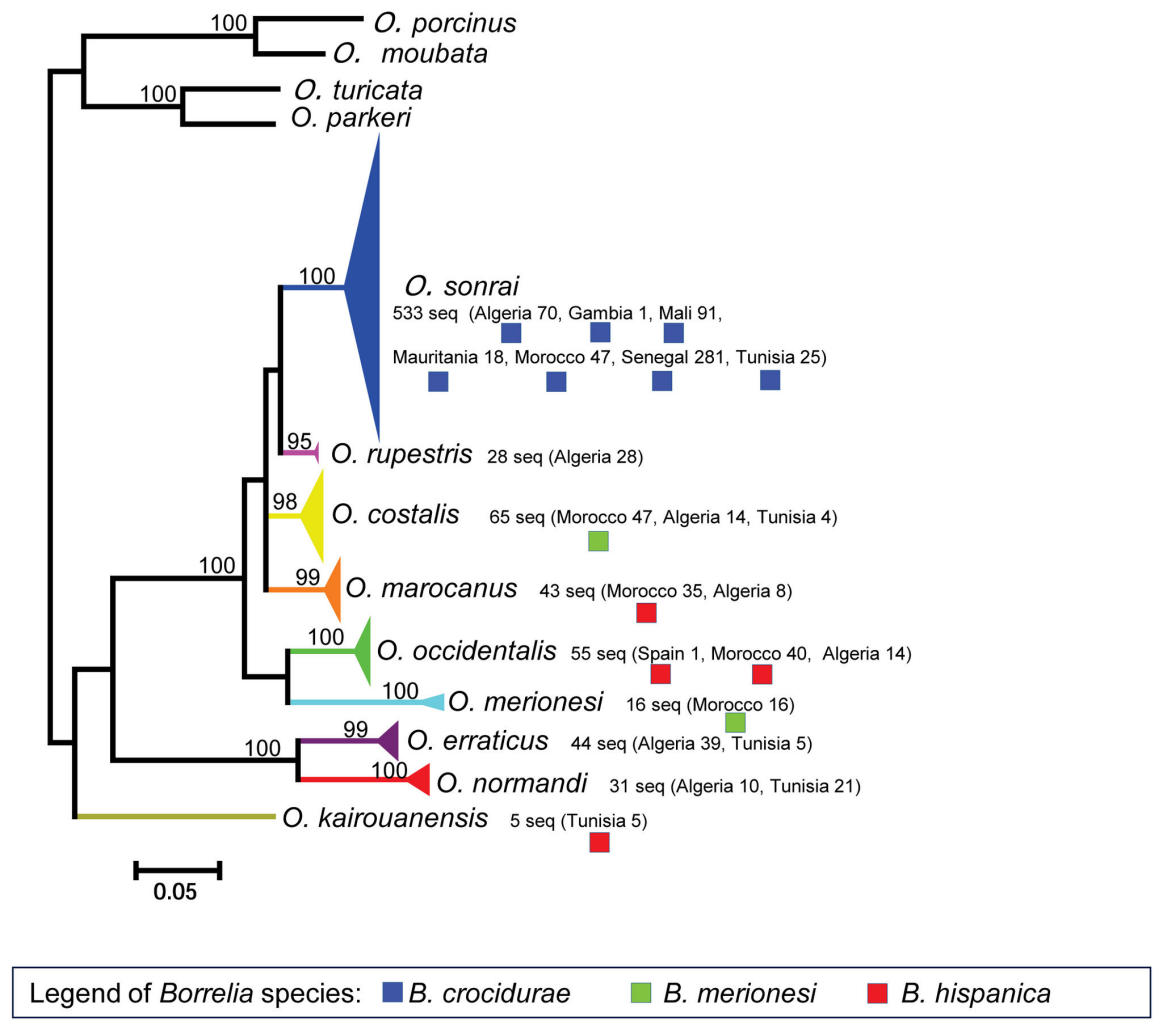
Phylogenetic relationships among *Ornithodoros* species using 16S rRNA sequences (820 seq.). The colored triangles indicate the genetic diversity of the sequences detected for each *Ornithodoros* species. The colored-spots correspond to the *Borrelia* species detected in each tick species. The phylogram was constructed using a maximum-likelihood method from partial 16S sequence data (457 nucleotides). Bootstrap values >90 are shown (Scale bar, 0.05 substitutions per site). *Ornithodoros moubata* (GenBank accession number AB073679), *O. porcinus* (GenBank acc. no. AB105451), *O. turicata* (GenBank acc. no. L34327), and *O. parkeri* (GenBank acc. no. EU009925) were treated as outgroups. Five of these species are newly described: *O. occidentalis*, *O. costalis*, *O. rupestris*, *O. kairouanensis*, and *O. merionesi*.

The phylogenetic analysis based on 820 16S rRNA sequences divided on 165 unique sequences which clustered in nine entities of *Ornithodoros* ticks (Figure 2). The genetic distance estimates (Kimura 2-parameters model) between entities varied from 0.062±0.013 (*O. costalis* - *O. marocanus* distance) to 0.251±0.030 (*O. merionesi* - *O. normandi* distance) (Table 4). The nine entities were genetically differentiated and hybrids between populations of these areas were known to be infertile [29], indicating that they should be considered as nine different species, including five previously undescribed species (see below and Appendix for designation of types, description of species and criteria for diagnosis): 

**Table 4:** Estimates of molecular distance (Kimura 2-parameter model) between the nine *Ornithodoros* species based on 165 sequences of 16S rRNA.

	*O. costalis*	*O. marocanus*	*O. occidentalis*	*O. merionesi*	*O. kairouanensis*	*O. erraticus*	*O. normandi*	*O. rupestris*
*O. sonrai*	0.073 (0.013)	0.075 (0.013)	0.095 (0.017)	0.120 (0.019)	0.200 (0.025)	0.219 (0.025)	0.222 (0.026)	0.058 (0.012)
*O. costalis*		0.062 (0.013)	0.084 (0.015)	0.113 (0.020)	0.194 (0.022)	0.212 (0.023)	0.220 (0.026)	0.052 (0.011)
*O. marocanus*			0.087 (0.014)	0.113 (0.019)	0.185 (0.023)	0.209 (0.023)	0.212 (0.026)	0.061 (0.011)
*O. occidentalis*				0.114 (0.019)	0.204 (0.026)	0.224 (0.025)	0.242 (0.028)	0.079 (0.015)
*O. merionesi*					0.203 (0.026)	0.230 (0.026)	0.251 (0.030)	0.114 (0.020)
*O. kairouanensis*						0.186 (0.025)	0.223 (0.028)	0.184 (0.023)
*O. erraticus*							0.109 (0.015)	0.201 (0.023)
*O. normandi*								0.198 (0.023)

Standard error estimates are shown in parenthesis and were obtained by a boostrap procedure (100 replicates).

• *O. occidentalis* Trape, Diatta, Durand & Renaud, sp. nov.

Holotype: IRSNB IG.32.280/004/1; ZooBank: urn:lsid:zoobank.org:act:DC0E7B5B-FBC3-4A7B-A223-77E0F2CFF2F5; GenBank: KC311536, KC311537.

• *O. costalis* Diatta, Bouattour, Durand, Renaud & Trape, sp. nov.

Holotype: IRSNB IG.32.280/005/1; ZooBank: urn:lsid:zoobank.org:act:43B277DD-7488-4277-8EC6-7AA9653882D0; GenBank: KC311531, KC311532.

• *O. rupestris* Trape, Bitam, Renaud & Durand, sp. nov.

Holotype: IRSNB IG.32.280/003/1; ZooBank: urn:lsid:zoobank.org:act:B76003D9-C807-4194-97C7-1FE7CFA3398D; GenBank: KC311545.

• *O. kairouanensis* Trape, Diatta, Bouattour, Durand & Renaud, sp. nov.

Holotype: IRSNB IG.32.280/002/1; ZooBank: urn:lsid:zoobank.org:act:DCC9185B-5FA8-494C-BF45-985A60478DC7; GenBank: KC311546.

• *O. merionesi* Trape, Diatta, Belghyti, Sarih, Durand & Renaud, sp. nov.

Holotype: IRSNB IG.32.280/001/1; ZooBank: urn:lsid:zoobank.org:act:8B64EB2B-0288-4488-9E39-94A2E3367FF5; GenBank: KC311538. 

The four other species identified were: *O. erraticus*, GenBank: KC311539, KC311540, KC311541, neotype: IRSNB 32.280/006; *O. marocanus*, GenBank: KC11533, KC311537, KC311535; neotype: IRSNB 32.280/007; *O. sonrai*, GenBank: KC311525, KC311526, KC311527, KC311528, KC311529, KC311530, neotype: IRSNB 32.280/007; and *O. normandi*, GenBank KC311542, KC311543, KC311544. 

**Figure 3:**
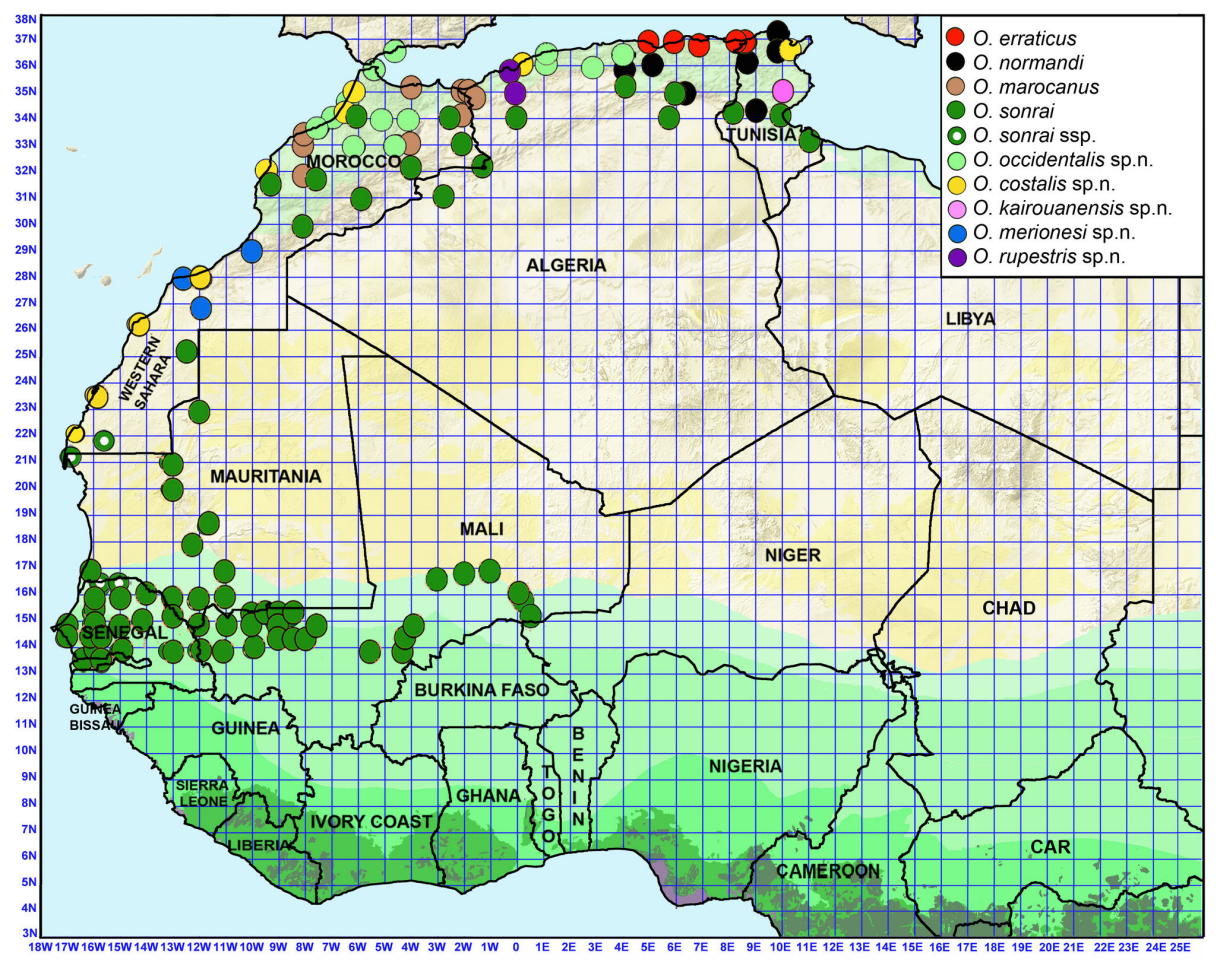
Map of northwestern Africa with the distribution of *Ornithodoros* species found in small mammal burrows.

Figure 3 shows that high specific diversity was observed in North Africa where the nine species were found and presented either a wide distribution in the most arid areas of Morocco, Algeria and Tunisia (one species: *O. sonrai*), or various restricted distribution patterns in coastal and/or inland areas of North Africa and Spain (eight species). 

 In West Africa, only *O. sonrai* was observed, and analysis performed on the 16S sequences of 533 *O. sonrai* ticks from 72 study sites in Sudanian, Sahelian and Saharan areas showed a large distribution of *O. sonrai* in West and North Africa (Figure 3). However, over the whole molecular range observed for this species, one group was genetically distinct (bootstrap >90). These ticks were sampled in an area extending from northwestern Senegal (Dagana, Richard-Toll) to northwestern Mauritania (Nouâdhibou) and the most southern part of Morocco (Adrar Souttouf) and we consider that they constitute a distinct sub-species of *O. sonrai* (Figure 3).

### Phylogenetic analysis of *Borrelia*

**Figure 4:**
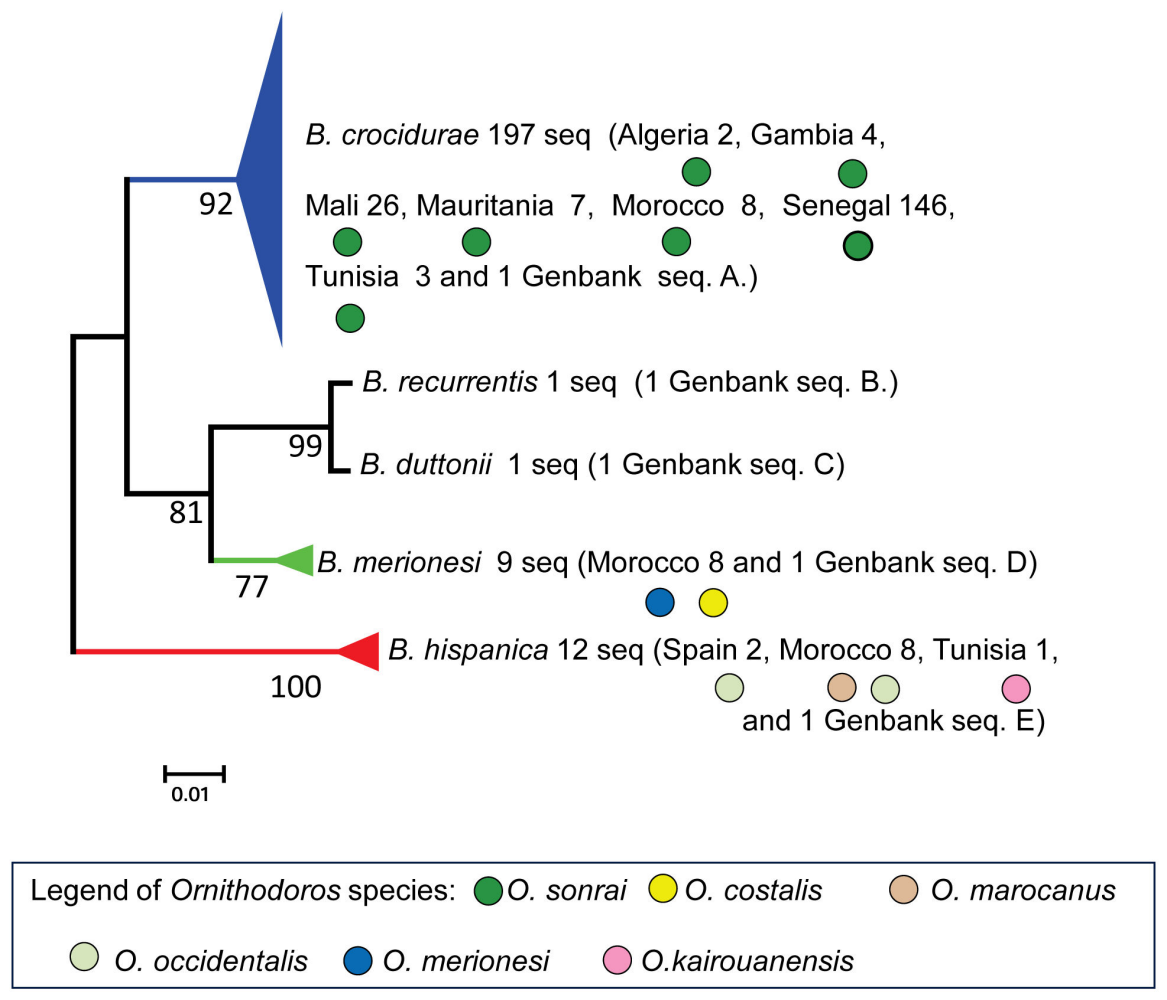
Phylogenetic relationships among *Borrelia* species using partial Intergenic spacer (IGS, 510 nucleotides) and partial FlaB gene (FLA, 269 nucleotides) concatenated sequences (PhyML 100 bootstraps, available at http://mobyle.pasteur.fr/cgi-bin/portal.py). The colored triangle estimated the *Borrelia* species diversity. We included in the *B. merionesi* clade (9 seq.) a *Borrelia* detected in a rodent captured in El Argoub (Morocco). The colored full circle correspond to the tick species determined by the 16S phylogenetic analysis of this study. The phylogram was constructed using a maximum-likelihood method from concatenated sequence data (220 sequences including GenBank reference sequences, 779 nucleotides). Bootstrap values >70 are shown (Scale bar, 0.01 substitutions per site). Seq. A.: *B. crocidurae* (GU350723 and NC017808), seq. B.: *B. recurrentis* (DQ000277 and DQ346814), seq. C.: *B. duttonii* (DQ000279 and DQ346833), seq. D.: *B. merionesi* (JX257047 and JX257050), seq. E.: *B. hispanica* (GU350718 and GU357614) and concatened sequences were used as references.

**Figure 5:**
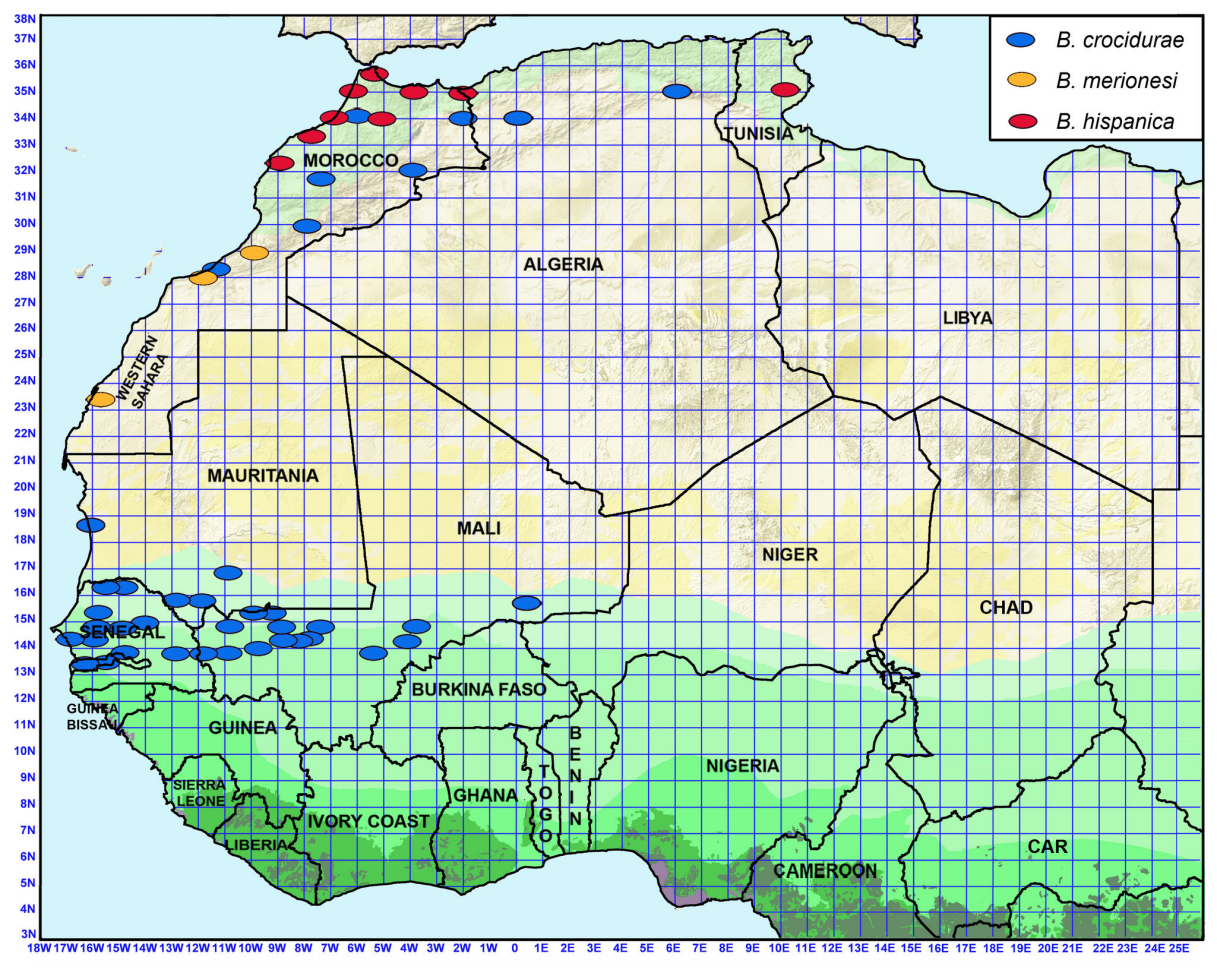
Map of northwestern Africa with the distribution of *Borrelia* species found in *Ornithodoros* ticks and/or rodents.

The phylogenetic analysis of *Borrelia* was based on 105 different sequences among 216 IGS-FLA sequences obtained. Figure 4 shows that three *Borrelia* species were present in sampled ticks: *B. crocidurae*, *B. hispanica*, and a third species, *B. merionesi*, a rare spirochaete that was formerly described from *Ornithodoros* ticks collected in the same area of southern Morocco than our specimens [59,60] (Figure 5). The genetic distance estimates among *Borrelia* species varied from 0.003±0.002 (*B. recurrentis* - *B. duttonii* distance) to 0.076±0.010 (*B. hispanica* - *B. recurrentis* distance) (Table 5). Sequences of *B. crocidurae* previously obtained from patients from Dielmo (Senegal) [39] and *B. hispanica* from patients from Kenitra, Morocco [30] were identical to those collected in *Ornothodoros* ticks in the same areas. 

**Table 5:** Estimates of molecular distance (Kimura 2-parameter model) between the three *Borrelia* species detected, based on 220 concatened IGS-FLA sequences (779 nucleotides), and comparison with *B. recurrentis* and *B. duttonii*.

	*B. crocidurae*	*B. hispanica*	*B. merionesi*	*B. recurrentis*
*B. crocidurae*	-			
*B. hispanica*	0.062 (0.008)	-		
*B. merionesi*	0.039 (0.007)	0.065 (0.009)	-	
*B. recurrentis*	0.043 (0.008)	0.076 (0.010)	0.035 (0.006)	-
*B. duttonii*	0.040 (0.007)	0.073 (0.010)	0.035 (0.006)	0.003 (0.002)

Standard error estimates are shown in parenthesis and were obtained by a bootstrap procedure (100 replicates).

 The IGS-FLA phylogenetic analysis indicated that *B. crocidurae* was only found in *O. sonrai* (196 out of 533 specimens, 36.7%), *B. hispanica* was found in *O. marocanus* (5/43, 11.6%), *O. occidentalis* (3/55, 5.5%) and *O. kairouanensis* (1/5, 20.0%), and *B. merionesi* was found in *O. costalis* (3/65, 4.6%) and *O. merionesi* (3/ 16, 18.8%). No *Borrelia* infection was detected in 28 specimens of *O. rupestris* from 2 study sites, nor in 44 specimens of *O. erraticus* from 5 study sites, nor in 31 specimens of *O. normandi* from 7 study sites.

### Diagnostic SNPs of *Ornithodoros* ticks

To allow a rapid molecular identification of *Ornithodoros* species, we highlighted 25 single nucleotide polymorphisms among the 9 species in 16S rRNA sequence dataset (Table 6). We detected at least one diagnostic SNP for each species, i.e. at a given position of the 16S sequence a nucleotide type is present in all sequences of a given species and absent in other species. According to species, the total number of diagnostic SNPs that can be used for taxonomic purpose of soft tick of the genus *Ornithodoros* varied from 1 to 8 (Table 6).

**Table 6:** Twenty-five Single Nucleotide Polymorphisms (SNPs) and their position which distinguish the nine *Ornithodoros* species, based on 820 16S rRNA aligned sequences (457 nucleotides).

Species	No. seq.				Diagnostic SNPs and their position on the 16S rRNA aligned sequences (457 nucleotides)																						Total SNPs
		54	59	74	81	96	111	137	157	172	181	189	194	209	214	217	237	244	256	257	258	288	294	301	405	438	
*O. sonrai*	534	A/G	G	A/C	A/G	A	T	T	**G**	A	T	A/T	T	C/T	T	A	T	T	A/C	A/C	A/C	A	T	A	A	A	1
*O. rupestris*	28	G	G	A	A	A	T	T	A	A	T	A	T	T	T	A	T	T	A	A	A	**G**	T	A	A	A	1
*O. costalis*	65	G	G	A	A	A	T	T	A	A	C/T	A	T	T	T	A	T	**A/C**	A	A	A	A	T	A	A	A	1
*O. marocanus*	43	G	**A**	A	A	A	T	T	A	A	T	A	T	G	T	A/G	T	T	A	A/T	A	A	T	A	A	A	1
*O. occidentalis*	55	G	G	A	A/G	A	T	T	A	**T**	T	A	A	G	**A**	A	T	T	A	A	A	A	T	A	A	A	2
*O. merionesi*	16	G	G	A	A	T	T	T	A	A	T	A	T	**A**	T	A	**A**	T	**T**	T	A	A	T	**G**	A	G	4
*O. erraticus*	44	G	G	**G**	A	A	**G**	T	A	A/G	**G**	A	**C**	**T**	T	A	T	T	A	A	**T**	A	**G**	A	**T**	A/G	8
*O. normandi*	31	**T**	G	A	A	**G**	A/T	**A**	A	A	**A**	A	A	T	T	G	T	T	A	A	A	A	A	A	G	A/C	4
*O. kairouanensis*	5	G	G	**T**	**T**	T	T	T	A	A	T	**C**	**G**	T	T	**T**	T	**G**	A	**G**	A	A	A	A	G	**T**	8

The nucleotide in bold underlined are the SNP diagnostic of the species considered. For example, in position 54 of the aligned sequences, the nucleotide T in bold underlined is present in all sequences of the *O. normandi* and absent in any sequences of other species. Other example, in position 244 the nucleotides A and C are diagnostic for *O. costalis*. No. seq.: number of analyzed sequences.

### Environmental factors and tick species distribution

**Figure 6:**
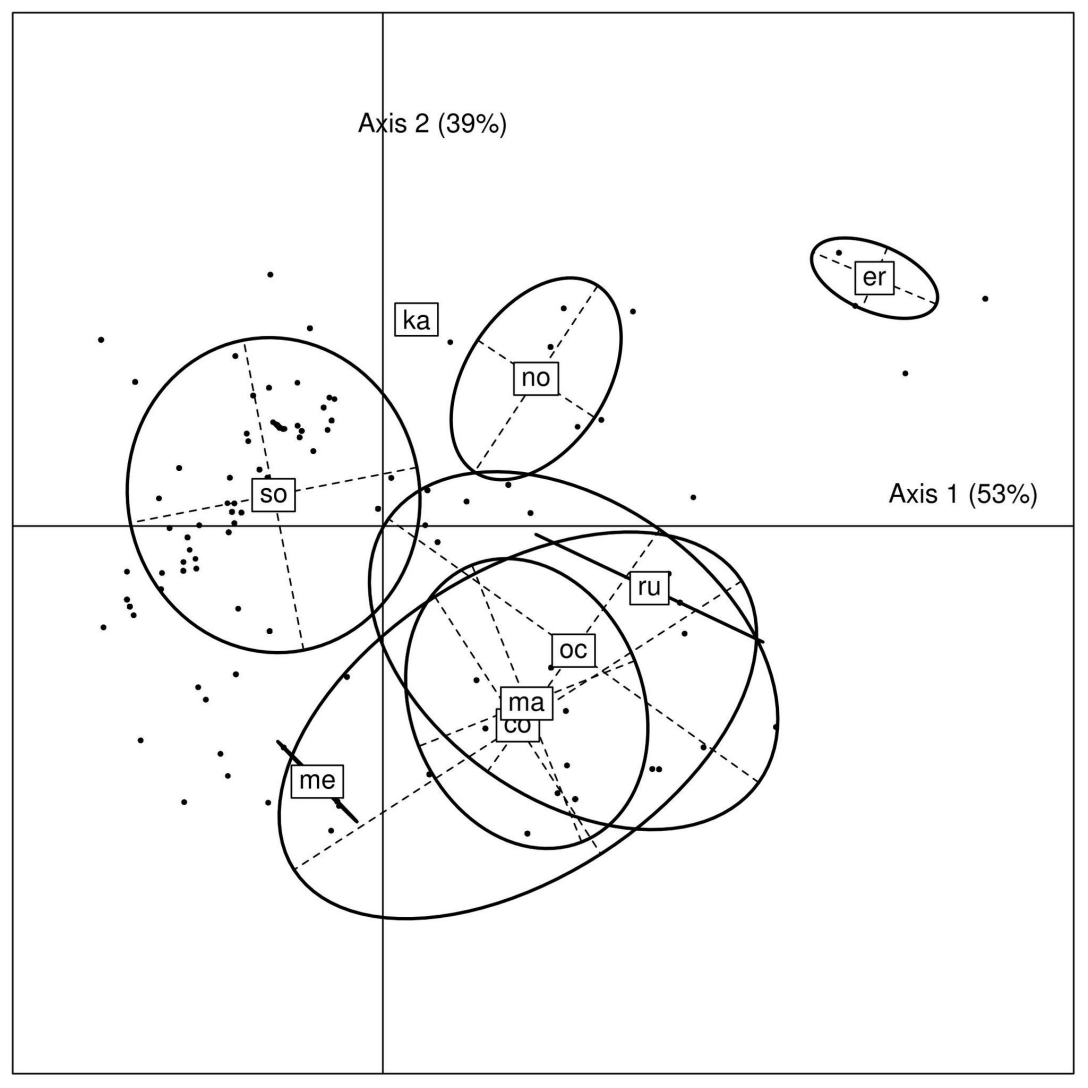
Projection of individual ticks on the first factorial plane of a Linear Discriminant Analysis. All ticks collected in the same location project to the same point. The percentages along the axes labels are the proportions of between-species variance explained by the corresponding linear discriminant. Units have no meaning. The labels indicating each species are located at the center of the individual points belonging to that species. Ellipses encompass around 66% of the distribution of each species. Abbreviations: so: *O. sonrai*, co: *O. costalis*, ma: *O. marocanus*, oc: *O. occidentalis*, me: *O. merionesi*, ka: *O. kairouanensis*, er: *O. erraticus*, no: *O*. *normandi*, ru: *O. rupestris*.

To investigate the relationship between environmental factors and species distribution, we included in the analysis ticks from 110 study sites. Figure 6 shows the projection of the individual ticks on the first factorial plane. The first two axes explain 92% of the between-species variance. The predictive power (percentage of well-classified data) is 76%. The variable most positively correlated with axis 1 is the mean annual rainfall (cor = 0.87), the most negatively correlated with axis 1 is mean temperature (cor = - 0.69). The variable most positively correlated with axis 2 is the longitude (cor = 0.81), and the most negatively correlated with axis 2 is elevation (cor = - 0.45). *O. sonrai* lies essentially on the negative part of axis 1, and thus appears related to drier climates and higher temperature than the other species. *O. costalis*, *O. marocanus*, *O. occidentalis* and *O. merionesi* lie generally on the negative part of axis 2 and are not well separated. *O. normandi* and *O. erraticus* lie on the positive part of both axes. Longitude (except for *O. costalis*) and rains (high rains for *O. erraticus* and *O. normandi*, low rains for *O. merionesi*) appear as the most discriminant factors.

## Discussion

 Genetic distances between the nine clades of *Onithodoros* ticks collected in this study ranged from 5.2 to 25.1%, a distance similar to those found between *Ornithodoros* species from other parts of the world. For comparison, the genetic distance between *O. puertoricensis* and *O. rioplatensis*, two species from South America, based on 16S rRNA sequencing, is approximately 12.7% [61]. Morphologically, it was easy to separate these nine species in two groups, the first one comprising two small species distributed in northeastern Algeria and northwestern Tunisia and characterized, among other diagnostic characters, by a transverse groove on coxae I, the second one comprising the seven other species and characterized by an oblique groove on coxae I, as previously illustrated elsewhere [31]. These two groups are confirmed by our molecular study (Figure 2). The two species of the first group includes respectively (a) all specimens from La Calle, Algeria - type locality of *O. erraticus* (Lucas, 1849) - and nearby coastal sites of Algeria and Tunisia, and (b) all specimens from Le Kef, Tunisia - type locality of *O. normandi* Larousse, 1923 - and nearby highland sites of Tunisia and Algeria [62,63]. None of 75 specimens of these two species were found infected by *B. hispanica*, *B. crocidurae* or *B. merionesi* in this study nor in a previous study in Tunisia [31], suggesting that *O. erraticus* sensu strico and *O. normandi* may be poor vectors or reservoirs of TBRF. By contrast, six of the seven species of the second morphological group of *Ornithodoros* ticks were found infected by one of these *Borrelia* species, most often with high prevalence rates (minimum 4.6% for *O. costalis*, maximum 36.6% for *O. sonrai*).

**Figure 7:**
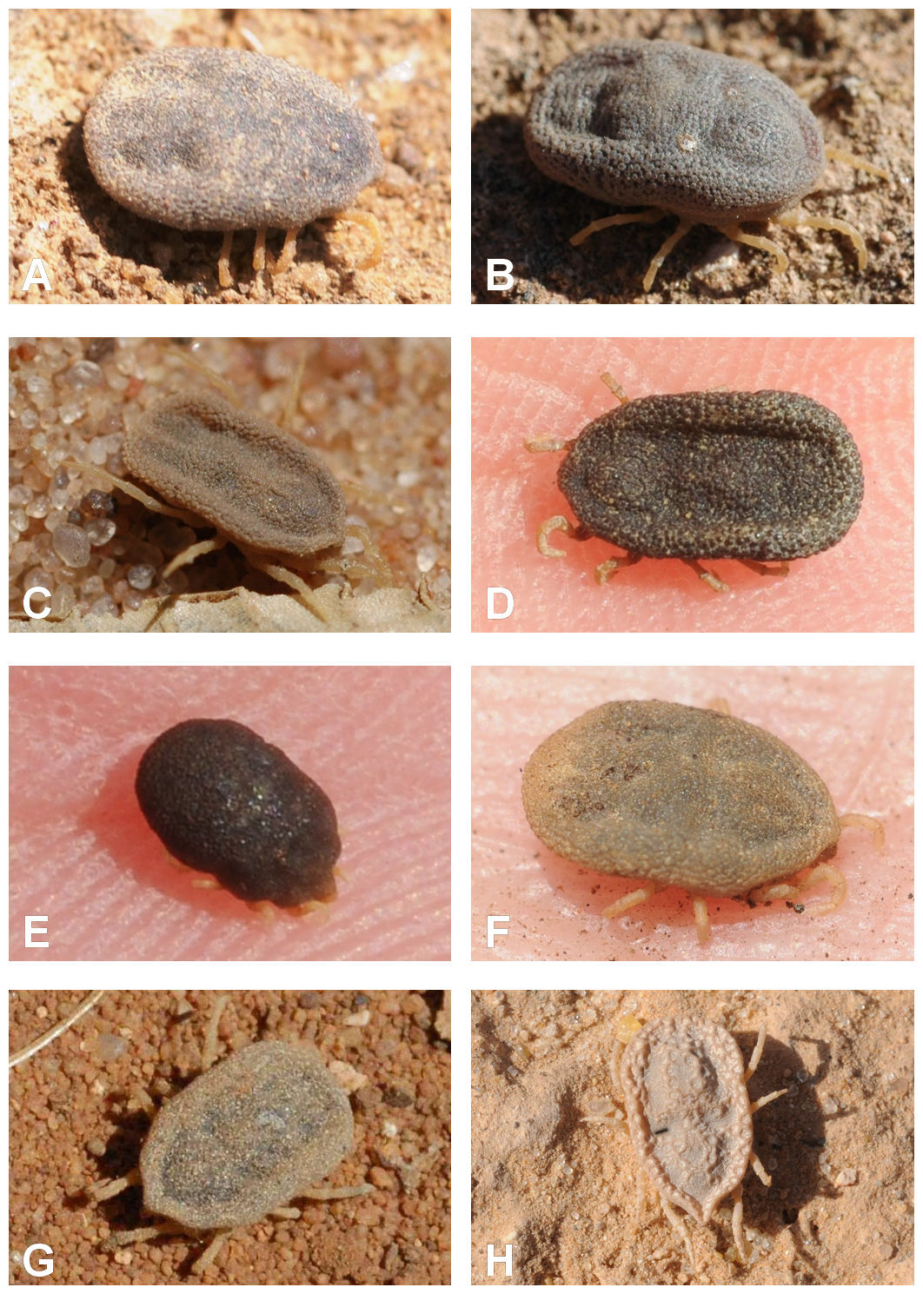
Dorsal view of *O. erraticus* (A), *O. marocanus* (B), *O. sonrai* (C), *O. costalis* (D), *O. merionesi* (E), *O. occidentalis* (F), *O. rupestris* (G), and *O. kairouanensis* (H).

 Among the seven species of the second group, only two species were previously known: *O. marocanus* Velu, 1919, type locality “vieille casbah” near Casablanca, Morocco and *O. sonrai* Sautet and Witkovski, 1943, type locality Gao, Mali [64,65]. However, since the work of Colas-Belcour [66], *O. marocanus* was considered as a junior synonym of *O. erraticus* [67,68]. Although most Algerian arthropods collected by Lucas [62] were deposited at the Museum National d’Histoire Naturelle in Paris, part of the collection was lost, including the three *O. erraticus* syntypes. The original description of these specimens was very short and *O. erraticus* was later redescribed by Neumann [69] on the basis of specimens not from La Calle but from Nemours (= Ghazaouet) and Marnia in northwestern Algeria near the Moroccan border, an area where our study shows that *O. erraticus* is absent but where *O. marocanus* is widely distributed. Since our study also shows that *O. erraticus* is the only species distributed in coastal areas of northeastern Algeria, from where the three syntypes of Lucas were described [61] and our specimens match their short description, *O. marocanus* must be resurrected from the synonymy of *O. erraticus* and the distribution of this later species appears much more restricted than previously thought. Morphologically, we failed to find good criteria for distinguishing *O. erraticus* from *O. normandi*, but these two species are clearly distinct genetically and have also distinct ranges and ecological preferences. In addition, comprehensive cross-mating experiments between eight populations of *O. erraticus* sensu lato from areas investigated in our study (Casablanca, Oujda, Marrakech, Tunis, Dakar, southern Morocco, southern Spain) and from central and southern Iran performed by Chabaud [29] in the early 1950s indicated that all hybrids of the first or second generation were either sterile or have very low fertility [29]. The results of these experiments combined with our data and the partial sympatry of several of these species clearly support the full specific rank of the seven species of the *O. marocanus* / *O. sonrai* group identified in our study (Figure 7).

 Interestingly, our data confirm the tick-spirochete species specificity highlighted by previous authors [70]. *B. crocidurae* was only detected in *O. sonrai*, and both this vector and this spirochete were genetically very close North and South of the Sahara desert, certainly because they present a continuous distribution through Mauritania and Western Sahara (but probably not through central Sahara, a much dryer area [57] where we failed to find *O. sonrai*). The only apparent disjunction in the distribution of this vector was observed in central Mali, where we failed to find a connection between the populations of *O. sonrai* bordering the Niger River and those distributed from western Mali to North Africa. *B. hispanica* was detected in *O. marocanus*, *O. occidentalis* and *O. kairouanensis*, a complex of three large species (female adult length 5.5 - 7.5 mm) distributed in typically Mediterranean areas of Morocco, Algeria, Tunisia and Spain and previously confused with *O. erraticus* [2,23,28-32,60,66-69]. Finally, the poorly known *B. merionesi* [59,60] appears restricted to Atlantic coastal areas of the Sahara desert where it is transmitted by two *Ornithodoros* species previously confused with *O. sonrai*: *O. costalis* and *O. merionesi*. Interestingly, no *Ornithodoros* species was found infected by more than one *Borrelia* species despite the high prevalence of infected ticks.

The considerable abundance of *Ornithodoros* TBRF vectors in North and West Africa - from 39.5 % of rodent burrows colonized by vector ticks in Morocco to 13.7 % in West Africa (Senegal, The Gambia, Mali and Mauritania) - coupled with the high infection rate of these vectors by at least two *Borrelia* species pathogenic for humans suggest that TBRF is a common disease in the whole range of distribution of these vectors. In rural Senegal, we have previously shown that the incidence of *B. crocidurae* TBRF reached 11 per 100 person-years and was the highest described in the world literature for any bacterial disease [39]. In northwestern Morocco, we found that 20.5% of patients with an unexplained fever had *B. hispanica* TBRF [30]. Clearly, similar incidence rates are likely to occur in most areas where we demonstrated the occurrence of one or more of the seven species of the *O. marocanus* - *O. sonrai* complex. It is not known if *B. merionesi* is also pathogenic for humans. However, a few previous experiments with this spirochete failed to induce TBRF in humans [59].

We consistently failed to find any *Ornithodoros tick* inside burrows or houses in West Africa south of 13°N and east of 01°E. We also failed to find these ticks in Cameroon and Chad. In the literature, there were in these areas only two mentions of *Ornithoros* species known as potential vectors of TBRF, one doubtful report of two larvae of *O. erraticus* collected on *Arvicanthis niloticus* in Ndjamena before 1959 [71] and the mention of one nymph of *O. sonrai* collected in a burrow of *Xerus erythropus* in Niamey in 1956 [23]. The later report was consistent with the current known distribution of *O. sonrai* along the Niger River in Mali, but our extensive investigations (132 burrows examined) in Niamey and along the Niger River between Niamey and the Malian border were negative. In Mali, a recent comprehensive study of the small mammal reservoir of *B. crocidurae* in 20 villages from different areas of the country indicated that 11% of 663 rodents and 14% of 63 shrews had antibodies to spirochetes and 2.2% had confirmed active *B. crocidurae* infections [37]. Interestingly, distribution of seropositive mammals and *O. sonrai* ticks supported our own data, except for some villages of southern Mali where a few rodents were found seropositive although collected south of the known distribution limit of *O. sonrai*. Since low rates of serological cross reactivity and/or dispersal or accidental introduction of rodents south of places where they were infected cannot be excluded, further studies are needed to confirm local transmission of TBRF in these areas. In the Malian study, three rodent species (*M. erythroleucus*, *M. natalensis*, *P. daltoni*) and two insectivores species (*C. olivieri*, *C. fulvastra*) were found infected by thin blood film examination, a method where the amount of blood examined in each microscopic field is approximately 20-fold less than by thick blood film examination [72]. In Senegal, additional mammal species found infected during previous studies include *A. albiventris*, *R. norvegicus*, *G. gambianus* and *G. gracilis* [35,36]. With these species and *C. fulvastra* found infected in Mali [37], the known small mammal reservoir of TBRF in West and North Africa currently comprises 21 species. 

A recent study in Togo indicated that 7.9% and 1.2% of 239 patients with fever from all regions of the country had *B. crocidurae* and *B. duttonii* infections, respectively, according to PCR blood testing [73]. However, in wet savanna and forest areas of West Africa, *Ornithodoros* ticks have never been reported [23,67,74,75], and this was confirmed by our surveys in Togo, Benin, Guinea, Guinea Bissau, Ivory Coast and Liberia where a total of 900 burrows and several hundred household examined were negative. If the occurrence of local transmission of *B. crocidurae* and *B. duttonii* in these areas is confirmed (since thick blood films failed to show the presence of spirochetes in PCR positive blood samples in the Togo study), this would suggest the existence of unknown vector / reservoir systems of *Borrelia* infections in wet savanna areas of West Africa.

Finally, our study as those previously conducted in northern, western, central, eastern and southern Africa [25,39,59,76,77] highlight the need of much more attention from health services, researchers and funding agencies to the continued burden of relapsing fevers due to *Borrelia* spp. in African populations.

## Appendix

### Description of *Ornithodoros merionesi*, *O. kairouanensis*, *O. rupestris*, *O. occidentalis* and *O. costalis*, and designation of neotypes for *O. erraticus*, *O. marocanus* and *O. sonrai.*

#### *Ornithodoros merionesi*

Trape, Diatta, Belghyti, Sarih, Durand & Renaud, sp. nov.

*Holotype*: IRSNB/RBINS IG.32.280/001/1, formerly IRD.TR-Mar7T8M, a nymph of 3.2 mm long collected on 6 October 2006 inside a rodent burrow in a steppe area located at 29°03’47”N / 09°55’42”W, approximately 16 km NE of Guelmin (Morocco), by G. Diatta, Y. Mané and J.F. Trape.

*Paratypes*: IRSNB/RBINS IG.32.280/001/2-14. Two females, two males and nine nymphs. Same date, location and collectors than the holotype.

*Diagnosis*: An *Argasidae tick* of the *O. marocanus* group of the genus *Ornithodoros*, characterized by an oblique groove on coxae I., and belonging to the subgroup of small / medium sized species (average female length < 5.2 mm, average male length <3.0 mm) of this complex (subgroup *O. sonrai* complex). Medium sized, morphologically indistinguishable from large specimens of *O. sonrai* and small specimens of *O. marocanus*, but genetically differing from these species and from all other species of the *O. marocanus* group by the unique position of A, A, T, and G on 16S rRNA aligned sequences (positions 209, 237, 256 and 301, respectively, see Table 6).

*Geographic distribution*: Morocco

#### *Ornithodoros kairouanensis*

Trape, Diatta, Bouattour, Durand & Renaud, sp. nov.

*Holotype*: IRSNB/RBINS IG.32.280/002/1, formerly IRD.TR-Tun5T3MN, a female of 7.1 mm long collected on 22 May 2010 inside a rodent burrow in a small cliff bordering a wadi located at 35°03’35”N / 10°02’23”E, approximately 70 km S of Kairouan (Tunisia), by G. Diatta and J.F. Trape.

*Paratypes*: IRSNB/RBINS IG.32.280/002/2-3. Two nymphs. Same date, location and collectors than the holotype.

*Diagnosis*: An Argasidae tick of the *O. marocanus* group of the genus *Ornithodoros*, characterized by an oblique groove on coxae I, and belonging to the subgroup of large species (average female length > 5.2 mm, average male length >3.0 mm) of this complex (subgroup *O. marocanus* complex). This species is characterized by crater-like formations on the soft dorsal cuticle (see Fig. 7H). Genetically differing from *O. marocanus* and from all other species of the *O. marocanus* group by the unique position of T, T, C, G, T, G, G, and T on 16S rRNA aligned sequences (positions 74, 81, 189, 194, 217, 244, 257 and 438, respectively, see Table 6).

*Geographic distribution*: Tunisia

#### *Ornithodoros rupestris*

Trape, Bitam, Renaud & Durand, sp. nov.

*Holotype*: IRSNB/RBINS IG.32.280/003/1, formerly IRD.TR-Alg12066bMN, a female of 6.1 mm long collected on 9 June 2012 inside a rodent burrow in a canyon located at 35°56’08”N / 00°05’07”E in the town of Mostaganem (Algeria) by J.F. Trape and I. Bitam.

*Paratypes*: IRSNB/RBINS IG.32.280/003/2-12. Three females, three males and five nymphs. Same date, location and collectors than the holotype.

*Diagnosis*: An Argasidae tick of the *O. marocanus* group of the genus *Ornithodoros*, characterized by an oblique groove on coxae I, and belonging to the subgroup of large species (average female length > 5.2 mm, average male length >3.0 mm) of this complex (subgroup *O. marocanus* complex). Morphologically indistinguishable from *O. marocanus*, but genetically differing from *O. marocanus* and from all other species of the *O. marocanus* group by the unique position of G on 16S rRNA aligned sequences (position 288, see Table 6). 

*Geographic distribution*: Algeria.

#### *Ornithodoros occidentalis*

Trape, Diatta, Durand & Renaud, sp. nov.

*Holotype*: IRSNB/RBINS IG.32.280/004/1, a female of 5.3 mm long collected on 18 July 2010 inside a rodent burrow in a pine woodland located at 36°38’25”N / 04°30’41”W near Torremolinos, Spain by G. Diatta and J.F. Trape. 

*Paratypes*: IRSNB/RBINS IG.32.280/004/2-3. Two females. Same date, location and collectors than the holotype.

*Diagnosis*: An *Argasidae tick* of the *O. marocanus* group of the genus *Ornithodoros*, characterized by an oblique groove on coxae I, and belonging to the subgroup of large species (average female length > 5.2 mm, average male length >3.0 mm) of this complex (subgroup *O. marocanus* complex). Morphologically indistinguishable from *O. marocanus*, but genetically differing from *O. marocanus* and from all other species of the *O. marocanus* group by the unique position of T and A on 16S rRNA aligned sequences (positions 172 and 214, respectively, see Table 6).

*Geographic distribution*: Spain, Morocco, Algeria.

#### *Ornithodoros costalis*

Diatta, Bouattour, Durand, Renaud & Trape, sp. nov.

*Holotype*: IRSNB/RBINS IG.32.280/005/1, formerly IRD.TR-Tun9T1MN, a female of 6.0 mm collected on 2 December 2010 inside a rodent burrow located at 36°36’42’’N, 10°10’14”E near Oudhna (Tunisia) by G. Diatta.

*Paratypes*: IRSNB/RBINS IG.32.280/005/2-4. Two females and one male. Same date, location and collectors than the holotype.

*Diagnosis*: An *Argasidae tick* of the *O. marocanus* group of the genus *Ornithodoros*, characterized by an oblique groove on coxae I., and belonging to the subgroup of large sized species (average female length > 5.2 mm, average male length >3.0 mm) of this complex (subgroup *O. marocanus* complex). Morphologically indistinguishable from *O. marocanus*, but genetically differing from these species and from all other species of the *O. marocanus* group by the unique position A/C on 16S rRNA aligned sequences (position 244, see Table 6).

*Geographic distribution*: coastal areas of Morocco, Algeria and Tunisia.

#### *Ornithodoros erraticus*

(Lucas, 1849).

The three syntypes of *Ornithodoros erraticus* are not in Paris MNHN. According to M. Judson (curator for arthropods, personal communication), they were certainly destroyed during transport to Paris as many other specimens of Lucas’s collection of Algerian arthropods. 

*Neotype*: IRSNB/RBINS 32.280/006, formerly IRD.TR-Alg5T5MN, a female of 3.1 mm long collected on 8 December 2009 inside a rodent burrow at 36°53’N / 08°31’E near La Calle, Algeria by G. Diatta. 

#### *Ornithodoros marocanus*

Velu, 1919.

The types of *O. marocanus* were sent by Velu [64] to the Pasteur Institute of Paris after he described this species. They could not be traced either in Paris or Casablanca and are now considered as lost (F. Rhodain, personal communication). 

*Neotype*: IRSNB/RBINS 32.280/007, formerly IRD.TR-Mar16T11ZC, a female of 5.9 mm long collected on 23 October 2009 inside a rodent burrow at 33°22’N / 08°00’W near Casablanca, Morocco by G. Diatta.

#### *Ornithodoros sonrai*

Sautet & Witkowski, 1943.

The types of *O. sonrai* could not be traced either in Paris or Dakar and are now considered as lost (J.-L. Camicas and F. Rodhain, personal communications).

*Neotype*: IRSNB/RBINS 32.280/008, formerly IRD.TR-MalG2T4, a female of 4.0 mm long collected on 22 January 2005 inside a rodent burrow at 15°59’22”N / 00°08’41”E in the village of Haousssa Foulane near Gao, Mali by G. Diatta, L. Vial & J.F. Trape.

## Supporting Information

Table S1 **Detailed results of *Ornithodoros* ticks surveys.**
(DOCX)

Table S2 **Detailed results of small mammals surveys.**
(DOCX)
