# Correction: The Epidemiology and Geographic Distribution of Relapsing Fever Borreliosis in West and North Africa, with a Review of the *Ornithodoros erraticus* Complex (Acari: Ixodida)

**DOI:** 10.1371/annotation/20b57909-df52-4073-a93f-a6689f84389d

**Published:** 2014-01-06

**Authors:** Jean-François Trape, Georges Diatta, Céline Arnathau, Idir Bitam, M’hammed Sarih, Driss Belghyti, Ali Bouattour, Eric Elguero, Laurence Vial, Youssouph Mané, Cellou Baldé, Franck Prugnolle, Gilles Chauvancy, Gil Mahé, Laurent Granjon, Jean-Marc Duplantier, Patrick Durand, François Renaud

The name of the 12th author was spelled incorrectly. The correct name is: Franck Prugnolle. 

