# Supplementary material for: The Epidemiology and Geographic Distribution of Relapsing Fever Borreliosis in West and North Africa, with a Review of the Ornithodoros erraticus Complex (Acari: Ixodida)
Source: PLoS One. 2013 Nov 4;8(11):e78473. doi: 10.1371/journal.pone.0078473 (PMC3817255; doi:10.1371/journal.pone.0078473)
Supplement: Table S1 — Detailed results of Ornithodoros ticks surveys. (DOCX) [file pone.0078473.s001.docx]

**Supplementary Table S1.** Detailed results of *Ornithodoros* ticks surveys

| **Study area** | **Coordinates of**  **sampling sites** | **Date** | **Burrows** | | ***Ornithodoros***  **species** |
| --- | --- | --- | --- | --- | --- |
|  |  |  | **Studied** | **With**  ***Ornithodoros*** |  |
| **Morocco** |  |  |  |  |  |
| Tetouan 1 | 35°52’N/05°21’W | 28/10/2009 | 10 (N)* | 7 (70%)* | *O. marocanus* |
| Tetouan 2 | 35°47’N/05°20'W | 28/10/2009 |  |  |  |
| Tetouan 3 | 35°51’N/05°20’W | 28/10/2009 |  |  |  |
| Izemmourèn | 34°11’N/03°59’W | 29/10/2009 | 15 (F) | 4 (27%) | *O. marocanus* |
| Berkane Oued Kiss | 34°59’N/02°08’W | 12/10/2006 | 30 (N) | 15 (50%) | *O. marocanus* |
| Ghouazi / Garba | 35°01’N/06°08’W | 12/05/2010 | 15 (F) | 15 (100%) | *O. costalis* |
| Kenitra 1 | 34°18’N/06°29’W | 10/10/2006 | 15 (N)* | 2 (13%)* | *O. costalis* |
| Kenitra 2 | 34°18’N/06°28’W | 10/10/2006 |  |  |  |
| Oued Ouerrha | 34°34’N/06°04’W | 30/10/2009 | 10 (F) | 5(50%) |  |
| Ouled Ziane 1 | 34°33’N/06°22’W | 31/10/2009 | 7 (PD)* | 1 (14%)* | Not tested |
| Ouled Ziane 2 | 34°32’N/06°20’W | 31/10/2009 |  |  |  |
| Rabat | 34°00’N/06°49’W | 12/05/2010 | 15 (N) | 13 (87%) | *O. occidentalis* |
| West Aiti Yadine | 34°00’N/06°02’W | 13/05/2010 | 15 (N/F) | 10 (67%) | *O. sonrai* |
| Fes (Diamant Vert) | 33°59’N/05°01’W | 14/05/2010 | 15 (N) | 9 (60%) | *O. occidentalis* |
| Beb-Lerba 1 | 34°00’N/04°05’W | 15/05/2010 | 8 (N)* | 1 (12%)* | *O. occidentalis* |
| Beb-Lerba 2 | 34°02’N/04°04’W | 15/05/2010 |  |  |  |
| Beb-Lerba 3 | 34°06’N/04°02’W | 15/05/2010 |  |  |  |
| Aïn-Benimathar (1) | 34°05’N/02°03’W | 13-14/10/2006 | 30 (N)* | 13 (43%)* | *O. marocanus, O. sonrai* |
| Aïn-Benimathar (2) | 34°07’N/02°02’W | 13-13/10/2006 |  |  |  |
| Oued Mellah | 33°39’N/07°23’W | 11/05/2010 | 15 (N) | 12 (80%) | *O. occidentalis* |
| Bir-Jdid | 33°22’N/08°00’W | 23/10/2009 | 15 (F; N) | 10 (67%) | *O. marocanus* |
| Oued Oum Er-Rbiat | 32°56’N/08°03’W | 18/05/2010 | 15 (N) | 12 (80%) | *O. marocanus* |
| Oued Grou 1 | 32°55’N/06°02’W | 13/05/2010 | 9 (N)* | 4 (44%)* | *O. occidentalis* |
| Oued Grou 2 | 32°55’N/06°03’W | 13/05/2010 |  |  |  |
| Oued Choufcherk | 33°02’N/04°00’W | 16/05/2010 | 15 (N) | 10 (67%) | *O. marocanus, O. occidentalis* |
| Tendrara 1 | 33°01’N/02°00’W | 14/10/2006 | 30 (N)* | 2 (7%)* | *O. sonrai* |
| Tendrara 2 | 33°08’N/01°58’W | 14/10/2006 |  |  |  |
| Figuig | 32°10’N/01°21’W | 15/10/2006 | 10 (N) | 3 (30%) | *O. sonrai* |
| Boudnib 1 | 31°59’N/03°59’W | 17/10/2006 | 30 (N)* | 4 (13%)* | *O. sonrai* |
| Boudnib 2 | 31°58’N/03°52’W | 17/10/2006 |  |  |  |
| Boudnib 3 | 31°58’N/03°55’W | 17/10/2006 |  |  |  |
| Marrakech | 31°44’N/07°58’W | 26/10/2009 | 15 (N) | 13 (87%) | *O. marocanus, O. sonrai* |
| Oued Tensift 1 | 32°00’N/09°20’W | 24/10/2009 | 16 (N/F)* | 8 (50%)* | *O. costalis, O. sonrai* |
| Oued Tensift 2 | 32°02'N/09°19'W | 24/10/2009 |  |  |  |
| Jbel Sarhro 1 | 30°56’N/05°50’W | 18-19/10/2006 | 30 (N)* | 8 (27%)* | *O. sonrai* |
| Jbel Sarhro 2 | 30°55’N/05°50’W | 18-19/10/2006 |  |  |  |
| Tata | 29°53’N/08°05’W | 07/10/2006 | 30 (N) | 14 (47%) | *O. sonrai* |
| Guelmin | 29°03’N/09°55’W | 06/10/2006 | 30 (N) | 14 (47%) | *O. merionesi* |
| Sidi Akhfennir | 27°57’N/11°57’W | 05/10/2006 | 30 (N) | 15 (50%) | *O. merionesi, O. costalis* |
| Sidi Ahmed 1 | 26°51’N/11°56’W | 04/10/2006 | 30 (N)* | 2 (7%)* | *O. merionesi* |
| Sidi Ahmed 2 | 26°57’N/11°57’W | 04/10/2006 |  |  |  |
| Sidi Ahmed 3 | 26°53’N/11°57’W | 04/10/2006 |  |  |  |
| Boujdour | 26°13’N/14°20’W | 01/10/2006 | 10 (N) | 2 (20%) | *O. costalis* |
| Bou Kra 1 | 25°58’N/12°52’W | 03/10/2006 | 30 (N)* | 0* |  |
| Bou Kra 2 | 26°02’N/12°50’W | 03/10/2006 |  |  |  |
| Galtat Zemmour | 25°13’N/12°26’W | 02/10/2006 | 20 (N) | 2 (10%) | *O. sonrai* |
| El Argoub | 23°33’N/15°53’W | 01/10/2006 | 10 (N) | 5 (50%) | *O. costalis* |
| Aousserd | 22°37’N/14°28’W | 13/11/2011 | 12 (N) | 1 (8%) | Not tested |
| Lahmiris | 22°04’N/16°35’W | 14/11/2011 | 6 (N) | 1 (17%) | *O. costalis* |
| Adrar Souttouf | 21°51’N/15°29’W | 16/11/2011 | 12 (N) | 2 (17%) | *O. sonrai* ssp. |
| ***Total Morocco*** |  |  | **605** | **239 (39.5%)** |  |
| **Algeria** |  |  |  |  |  |
| Vieille Calle 1 | 36°54’N/08°20’E | 08-10/12/2009 | 23 (N)* | 8 (35%)* | *O. erraticus* |
| Vieille Calle 1 | 36°53’N/08°24’E | 09/10/12/2009 |  |  |  |
| Vieille Calle 2 | 36°53’N/08°31’E | 08-10/12/2009 |  |  |  |
| Vieille Calle 3 | 36°54’N/08°31’E | 08-10/12/2009 |  |  |  |
| Vieille Calle 4 | 36°53’N/08°34’E | 09-10/12/2009 |  |  |  |
| Salah Bouchaour | 36°41’N/06°51’N | 06/06/2010 | 5 (N) | 5 (100%) | *O. erraticus* |
| Taher 1 | 36°47’N/05°54’E | 31/05/2010 | 10 (N)* | 2 (20%)* | *O. erraticus* |
| Taher 2 | 36°49’N/05°55’E | 31/05/2010 |  |  |  |
| Taher 3 | 36°48’N/05°54’E | 31/05/2010 |  |  |  |
| Taya 1 | 36°00’N/05°58’E | 15/05/2009 | 45 (N)* | 0* |  |
| Taya 2 | 35°48’N/06°06’E | 14/05/2009 |  |  |  |
| Taya 3 | 35°55’N/05°52’E | 05/06/2010 |  |  |  |
| M’Chounèche 1 | 34°55’N/05°58’E | 11/05/2009 | 36 (N)* | 19 (53%)* | *O. sonrai, O. normandi* |
| M’Chounèche 2 | 34°57’N/06°00’E | 11/05/2009 |  |  |  |
| M’Chounèche 3 | 34°56’N/05°58’E | 18/05/2009 |  |  |  |
| M’Chounèche 1 2010 | 34°55’N/05°58’E | 11/05/2010 |  |  |  |
| Oued Melah 1 | 33°56’N/05°42’E | 13/05/2009 | 33 (N)* | 3 (9%)* | *O. sonrai* |
| Oued Melah 2 | 34°00’N/05°52’E | 13/05/2009 |  |  |  |
| Oued Melah 1 2010 | 33°56’N/05°42’E | 22/10/2010 |  |  |  |
| Sidi Madhi/Sebseb 1 | 33°03’N/06°06’E | 12/05/2009 | 30 (N)* | 0* |  |
| Sidi Madhi/Sebseb 2 | 32°10’N/03°33’E | 20/06/2011 |  |  |  |
| Sidi Madhi/Sebseb 3 | 32°05’N/03°46’E | 20/06/2011 |  |  |  |
| Oued Abani | 27°39’N/00°20’W | 22/06/2011 | 30 (N) | 0 |  |
| Taghit 1 | 31°02’N/02°11’W | 23/06/2011 | 12 (N)* | 0* |  |
| Taghit 2 | 31°10’N/02°16’W | 24/06/2012 |  |  |  |
| Abadla 1 | 30°59’N/02°43’W | 24/06/2012 | 14 (N)* | 1 (7%)* | *O. sonrai* |
| Abadla 2 | 31°05’N/02°47’W | 24/06/2012 |  |  |  |
| Oued Saket 1 | 36°49’N/04°56’E | 02/06/2010 | 24 (N/F)* | 1 (4%)* | *O. erraticus* |
| Oued Saket 2 | 36°45’N/05°05’E | 01/06/2010 |  |  |  |
| Oued Saket 3 | 36°46’N/05°05’E | 01/06/2010 |  |  |  |
| Oued Saket 4 | 36°47’N/05°00’E | 02/06/2010 |  |  |  |
| Oued Saket 5 | 36°42’N/05°00’E | 02/06/2010 |  |  |  |
| Sétif 1 | 35°59’N/05°04’E | 03/06/2010 | 17 (N)* | 1 (6%)* | *O. normandi* |
| Sétif 2 | 36°12’N/05°30’E | 05/06/2010 |  |  |  |
| Bouira | 36°25’N/03°57’E | 07/06/2010 | 8 (N) | 5 (63%) | *O. occidentalis* |
| Melouza 1 | 35°51’N/04°06’E | 04/06/2010 | 10 (N)* | 2 (20%)* | *O. normandi* |
| Melouza 2 | 35°54’N/04°06’E | 04/06/2010 |  |  |  |
| Oued Magtaa | 35°09’N/04°06’E | 19/10/2010 | 7 (N) | 4 (57%) | *O. sonrai* |
| El Mesrane 1 | 34°56’N/03°06’E | 20-21/12/2010 | 30 (N)* | 0* |  |
| El Mesrane 2 | 35°03’N/03°02’E | 20-21/12/2010 |  |  |  |
| El Mesrane 3 | 35°06’N/03°01’E | 20-21/12/2010 |  |  |  |
| Berrouaghia 1 | 36°09’N/02°58’E | 09/06/2010 | 15 (N)* | 3 (20%)* | *O. occidentalis* |
| Berrouaghia 2 | 35°57’N/02°50’E | 09/06/2010 |  |  |  |
| Chlef | 36°05’N/01°06’E | 10/06/2010 | 5 (N) | 4 (80%) | *O. occidentalis* |
| Ténès | 36°28’N/01°07’E | 11/06/2010 | 6 (N) | 1 (17%) | *O. occidentalis* |
| Ghazaouet | 35°03’N/01°50’W | 04/06/2012 | 6 (N) | 2 (33%) | *O. marocanus* |
| Beni-Bahdel | 34°42’N/01°31’W | 03/06/2012 | 5 (N) | 1 (20%) | *O. marocanus* |
| Asla | 33°00’N/00°08’W | 05/06/2012 | 35 (N) | 0 |  |
| Bougtob | 34°00’N/00°02’E | 06/06/2012 | 8 (N) | 4 (50%) | *O. sonrai* |
| Saïda Mt Daïa 1 | 34°56’N/00°05’W | 08/06/2012 | 10 (N)* | 2 (50%)* | *O. rupestris* |
| Saïda Mt Daïa 2 | 34°48’N/00°03’E | 08/06/2012 |  |  |  |
| Mostaganem 1 | 35°56’N/00°05’E | 09/06/2012 | 6 (N) | 4 (67%) | *O. rupestris* |
| Mostaganem 2 | 35°58’N/00°08’E | 10/06/2012 | 5 (N) | 2 (40%) | *O. costalis* |
| ***Total Algeria*** |  |  | **435** | **74 (17.0%)** |  |
| **Tunisia** |  |  |  |  |  |
| Bizerte 1 | 37°19’N/09°50’E | 22/05/2010 | 12 (N)* | 1 (13%)* | *O. normandi* |
| Bizerte 2 | 37°14’N/09°43’E | 22/05/2010 |  |  |  |
| Bizerte 3 | 37°01’N/09°43’E | 21/05/2010 |  |  |  |
| Bizerte 4 | 37°00’N/09°44’E | 21/05/2010 |  |  |  |
| Bizerte 5 | 37°19’N/09°51’E | 22/05/2010 |  |  |  |
| Tabarka 1 | 36°51’N/08°43’E | 22/05/2010 | 6 (N)* | 2 (33%)* | *O. erraticus* |
| Tabarka 2 | 36°57’N/08°47’E | 22/05/2010 |  |  |  |
| Oudhna (Tunis) | 36°36’N/10°10’E | 02/12/2010 | 3 (F) | 2 (67%) | *O. normandi, O. costalis* |
| Le Kef | 36°07’N/08°38’E | 23/05/2010 | 6 (N) | 4 (67%) | *O. normandi* |
| Kairouan Sud | 35°03’N/10°02’E | 23/05/2010 | 10 (N) | 6 (60%) | *O. kairouanensis* |
| Oued Ramel | 34°07’N/09°58’E | 29/11/2010 | 8 (N) | 3 (38%) | *O. sonrai* |
| Tozeur 1 | 34°12’N/08°12’E | 01/12/2010 | 17 (N)* | 2 (12%)* | *O. sonrai* |
| Tozeur 2 | 34°00’N/08°10’E | 01/12/2010 |  |  |  |
| Tozeur 3 | 34°01’N/08°12’E | 01/12/2010 |  |  |  |
| Tozeur 4 | 34°06’N/08°15’E | 01/12/2010 |  |  |  |
| Tozeur 5 | 34°09’N/08°16’E | 01/12/2010 |  |  |  |
| Ben Guerdane 1 | 33°08’N/11°02’E | 30/11/2010 | 18 (N)* | 2 (11%)* | *O. sonrai* |
| Ben Guerdane 2 | 33°08’N/11°05’E | 30/11/2010 |  |  |  |
| Ben Guerdane 3 | 33°07’N/11°04’E | 30/11/2010 |  |  |  |
| Gafsa | 34°16’N/09°04’E | 04/10/2010 | 2 (N) | 1 (50%) | *O. normandi* |
| ***Total Tunisia*** |  |  | **82** | **23 (28.1%)** |  |
| **Mauritania** |  |  |  |  |  |
| Mbalou 1 | 15°01’N/12°04’W | 15/11/2003 | 30 (N)* | 17 (57%)* | *O. sonrai* |
| Mbalou 2 | 15°02’N/12°05’W | 15/11/2003 |  |  |  |
| Soufa 1 | 15°56’N/12°01’W | 13/11/2003 | 30 (N/HD)* | 8 (27%)* | *O. sonrai* |
| Soufa 2 | 15°56’N/12°04’W | 13/11/2003 |  |  |  |
| Soufa 3 | 15°58’N/12°04’W | 13/11/2003 |  |  |  |
| Soufa 4 | 15°58’N/12°05’W | 13/11/2003 |  |  |  |
| Soufa 5 | 15°55’N/12°02’W | 14/11/2003 |  |  |  |
| Iguéva 1 | 17°00’N/11°57’W | 17/11/2003 | 32 (N)* | 0* | *O. sonrai* |
| Iguéva 2 | 17°05’N/12°03’W | 17/11/2003 |  |  |  |
| Nbeïka 2003 | 17°59’N/12°14’W | 18-19/11/2003 | 27 (N/PD) | 11 (41%) | *O. sonrai* |
| Nbeïka 2004 | 17°59’N/12°14’W | 14-16/10/2004 | 30 (N) | 18 (60%) | *O. sonrai* |
| Rachid / Ag Mouret 1 | 18°47’N/11°41’W | 20/11/2003 | 56 (N)* | 1 (1.8%)* | *O. sonrai* |
| Rachid / Ag Mouret 2 | 18°53’N/11°49’W | 21/11/2003 |  |  |  |
| Rachid / Ag Mouret 3 | 19°11’N/11°55’W | 05/12/2007 |  |  |  |
| Rachid / Ag Mouret 4 | 18°55’N/11°48’W | 05/12/2007 |  |  |  |
| Oujeft | 20°00’N/13°03’W | 21/11/2003 | 30 (N/PD) | 2 (7%) | *O. sonrai* |
| Aggui 1 | 21°01’N/13°07’W | 26-27/11/2003 | 31 (N) | 1 (3%) | *O. sonrai* |
| Aggui 2 | 21°10’N/13°06’W | 26/11/2003 |  |  |  |
| Touajil 1 | 22°07’N/12°41’W | 28/11/2003 | 30 (N)* | 0* | *O. sonrai* |
| Touajil 2 | 22°02’N/12°49’W | 28/11/2003 |  |  |  |
| Guelb el Dlim 1 | 22°59’N/12°00’W | 30/11/2003 | 36 (N)* | 1 (3%)* | *O. sonrai* |
| Guelb el Dlim 2 | 22°55’N/12°09’W | 30/11/2003 |  |  |  |
| Araguib el Jahfa | 19°47’N/12°14’W | 12/10/2004 | 13 (N) | 0 |  |
| Nouadhibou | 21°16’N/16°50’W | 25/11/2005 | 6 (N) | 2 (33%) | *O. sonrai* ssp. |
| Azougui | 20°34’N/13°07’W | 03/12/2007 | 6 (N) | 0 |  |
| Tiouilît 1 | 18°55'N/16°10'W | 26/11/2007 | 30 (N)* | 0* |  |
| Tiouilît 2 | 18°52'N/16°09'W | 26/11/2007 |  |  |  |
| Tiouilît 3 | 18°36’N/16°04'W | 26/11/2007 |  |  |  |
| Tafolli | 18°45’N/15°34’W | 27/11/2007 | 8 | 0 |  |
| Nouakchott | 18°00’N/16°01’W | 20-21/04/2007 | 30 (N) | 0 |  |
| Ganeb 1 | 18°29’N/10°59’W | 07/12/2007 | 30 (N)* | 0* |  |
| Ganeb 2 | 18°27’N/10°50’W | 07/12/2007 |  |  |  |
| Makhrouga Ouest | 18°21’N/08°58’W | 09/12/2007 | 30 (N) | 0 |  |
| Enji 1 | 18°03’N/08°01’W | 10/12/2007 | 30 (N)* | 0* |  |
| Enji 2 | 17°59’N/08°01’W | 10/12/2007 |  |  |  |
| Ibibi Oglat | 17°00’N/16°05’W | 19-20/04/2007 | 30 (N) | 8 (27%) | *O. sonrai* |
| Amridjiel | 17°00’N/10°59’W | 03-04/12/2006 | 30 (N) | 15 (50%) | *O. sonrai* |
| Agerkaya-2 | 16°10’N/13°00’W | 16/12/2006 | 30 (N) | 11 (37%) | *O. sonrai* |
| Sallem 1 | 16°05’N/11°00’W | 06/12/2006 | 30 (N)* | 1 (3%)* | *O. sonrai* |
| Sallem 2 | 16°04’N/11°00’W | 06/12/2006 |  |  |  |
| Sallem 3 | 16°04’N/11°02’W | 06/12/2006 |  |  |  |
| El Beyyed 1 | 16°53’N/09°59’W | 14-15/03/2007 | 30 (HD/PD/N)* | 0* |  |
| El Beyyed 2 | 16°53’N/10°00’W | 14-15/03/2007 |  |  |  |
| Te-Nguembou | 16°03’N/09°55’W | 16/03/2007 | 30 (N) | 0 |  |
| Banète 1 | 15°51’N/09°06’W | 17-18/03/2007 | 30 (N)* | 0* |  |
| Banète 2 | 15°50’N/09°06’W | 17-18/03/2007 |  |  |  |
| Aïn-Biré 1 | 16°08’N/08°06’W | 19-20/03/2007 | 30 (N/PD)* | 0* |  |
| Aïn-Biré 2 | 16°09’N/08°06’W | 20/03/2007 |  |  |  |
| Archane 1 | 16°00’N/07°28’W | 29-30/03/2007 | 30 (HD/PD/N/F)* | 0* |  |
| Archane 2 | 16°00’N/07°29’W | 29-30/03/2007 |  |  |  |
| Chgueig 1 | 16°01’N/07°06’W | 21/03/2007 | 30 (PD/HD/N)* | 0* |  |
| Chgueig 2 | 16°01’N/07°05’W | 21-22/03/2007 |  |  |  |
| Chgueig 3 | 16°02’N/07°05’W | 21-22/03/2007 |  |  |  |
| Chgueig 4 | 16°02’N/07°06’W | 21-22/03/2007 |  |  |  |
| Chgueig 5 | 16°04’N/07°07’W | 22/03/2007 |  |  |  |
| Emmat Lakarich 1 | 15°59’N/06°30’W | 23-24/03/2007 | 30 (N/HD/PD)* | 0* |  |
| Emmat Lakarich 2 | 15°59’N/06°29’W | 23-24/03/2007 |  |  |  |
| Lambasite | 15°51’N/06°07’W | 25-26/03/2007 | 30 (N) | 0 |  |
| Lekrysse 1 | 15°30’N/07°01’W | 27-28/03/2007 | 30 (N/HD/PD)* | 0* |  |
| Lekrysse 2 | 15°30’N/07°00’W | 27-28/03/2007 |  |  |  |
| ***Total Mauritania*** |  |  | **905** | **96 (10.6%)** |  |
| **Senegal** |  |  |  |  |  |
| Keur Ngor | 13°58’N/16°01’W | 10/05/2003 | 30 (F/HD) | 6 (20%) | *O. sonrai* |
| Maka Yop | 14°01’N/15°01’W | 15-16/09/2005 | 30 (HD) | 17 (57%) | *O. sonrai* |
| Kalbirom 1 | 13°59’N/14°01’W | 08/05/2003 | 60 (N)* | 0* |  |
| Kalbirom 2 | 13°59’N/14°00’W | 08/05/2003 |  |  |  |
| Kalbirom 3 | 13°53’N/14°06’W | 09/05/2003 |  |  |  |
| Kalbirom 4 | 13°53’N/14°07’W | 09/05/2003 |  |  |  |
| Kéniéba 1 | 14°05’N/12°03’W | 06/05/2003 | 30 (N/HD)* | 13 (53%)* | *O. sonrai* |
| Kéniéba 2 | 14°06’N/12°03’W | 07/05/2003 |  |  |  |
| Kouthia | 14°04’N/13°08’W | 17/09/2005 | 30 (HD) | 12 (40%) | *O. sonrai* |
| Khor (NW Rosso) | 16°30’N/15°55’W | 18-19/06/2003 | 60 (N) | 0 |  |
| Taouey (Richard-Toll) | 16°27’N/15°42’W | 20-21/06/2003 | 30 (HD) | 11 (37%) | *O. sonrai* ssp. |
| Keur Momar Sarr 1 | 15°55’N/15°57’W | 01/07/2002 | 36 (N/F)* | 6 (17%)* | *O. sonrai* |
| Keur Momar Sarr 2 | 16°10’N-16°08’W | 01/08/2002 |  |  | *O. sonrai* |
| Keur Momar Sarr 3 | 15°58’N/15°55’W | 30/06/2002 |  |  |  |
| Khanène Khar | 15°30’N/16°01’W | 15-16/06/2003 | 30 (N/HD) | 27 (90%) | *O. sonrai* |
| Thianène 1 | 15°04’N/16°00’W | 28/06/2002 | 35 (N/F)* | 9 (26%)* | *O. sonrai* |
| Thianène 2 | 15°06’N/16°00’W | 28/06/2002 |  |  |  |
| Ina | 14°30’N/16°01’W | 13-14/06/2003 | 30 (HD/PD/N) | 23 (77%) | *O. sonrai* |
| Colobane 1 | 14°38’N/15°42’W | 26/06/2002 | 40 (N/PD)* | 6 (15%)* | *O. sonrai* |
| Colobane 2 | 14°36’N/15°41’W | 27/06/2002 |  |  |  |
| Kossanto 1 | 13°08’N/11°57’W | 18-20/0/2004 | 60 (HD/PD/N/F)* | 0* |  |
| Kossanto 2 | 13°08’N/11°58’W | 19/0/2004 |  |  |  |
| Dielmo 2002 | 13°43’N/16°24’W | 04/10/2002 | 342 (HD/PD) | 54 (16%) | *O. sonrai* |
| Dielmo 2003 | 13°43’N/16°24’W | 17-19/05/2003 | 100 (N/F) | 8 (8%) | *O. sonrai* |
| Dielmo 2012 | 13°43’N/16°24’W | 23/01/2012 | 25 (HD) | 10 (40%) | *O. sonrai* |
| NDiop 2002 | 13°41’N/16°22’W | 30-31/08/2002 | 50 (HD) | 0 |  |
| NDiop 2003 | 13°41’N/16°22’W | 12-13/05/2003 | 50 (F) | 1 (2%) | *O. sonrai* |
| NDiop 2012 | 13°41’N/16°22’W | 22/01/2012 | 30 (HD) | 9 (30%) | *O. sonrai* |
| Keur Aliou Gueye | 13°47’N/16°24’W | 23-24/06/2003 | 75 (HD/PD/F) | 3 (4%) | *O. sonrai* |
| Keur Mama Lamine | 13°46’N/16°23’W | 25-26/06/2003 | 50 (HD/PD) | 1 (2%) | *O. sonrai* |
| Nioro Alassane Tall | 13°46’N/16°20’W | 27-28/06/2003 | 47 (HD/PD) | 5 (11%) | *O. sonrai* |
| Bani | 13°45’N/16°28’W | 25-27/10/2002 | 50 (HD/PD) | 27 (54%) | *O. sonrai* |
| Firdaossi | 13°45’N/16°27’W | 23-24/10/2002 | 44 (HD/PD) | 19 (43%) | *O. sonrai* |
| Néma Ba | 13°44’N/16°28’W | 20-22/10/2002 | 50 (HD/PD) | 29 (58%) | *O. sonrai* |
| Santamba | 13°44’N/16°25’W | 17-18/1020/02 | 50 (HD/PD) | 2 (4%) | *O. sonrai* |
| Keur Lahine Socé 2002 | 13°44’N/16°25’W | 19-20/10/2002 | 41 (HD/PD) | 5 (12%) | *O. sonrai* |
| Keur Lahine Socé 2003 | 13°44’N/16°25’W | 20-1/05/2003 | 50 (N/F) | 11 (22%) | *O. sonrai* |
| Keur Lahine Fatim | 13°44’N/16°24’W | 24-25/08/2002 | 50 (HD/PD) | 0 |  |
| Sabouya | 13°43’N/16°25’W | 15-16/10/2002 | 50 (HD/PD) | 19 (38%) | *O. sonrai* |
| Touba-Nding | 13°43’N/16°25’W | 13-14/10/2002 | 50 (HD/PD) | 10 (20%) | *O. sonrai* |
| Néma-Nding | 13°42’N/16°25’W | 11-13/10/2002 | 50 (HD/PD) | 1 (2%) | *O. sonrai* |
| Médina Santhie 2002 | 13°42’N/16°24’W | 26-27/08/2002 | 44 (HD/PD) | 1 (2%) | *O. sonrai* |
| Médina Santhie 2003 | 13°42’N/16°24’W | 13-15/05/2003 | 51 (N/F) | 1 (2%) | *O. sonrai* |
| Passi Nenderling 2002 | 13°42’N/16°23’W | 28-29/08/2002 | 50 (HD/PD) | 1 (2%) | *O. sonrai* |
| Passi Nenderling 2003 | 13°42’N/16°23’W | 13-15/05/2003 | 50 (N/F) | 3 (6%) | *O. sonrai* |
| Same | 13°37’N/16°25’W | 08-09/07/2003 | 50 (HD/PD) | 0 |  |
| Sirmang | 13°37’N/16°23’W | 10-11/08/2003 | 50 HD/PD) | 0 |  |
| Keur Moussa Séni | 13°37’N/16°21’W | 12-15/07/2003 | 50 (HD/PD) | 0 |  |
| Madina Djikoye | 13°37’N/16°17’W | 13-15/09/2002 | 50 (HD/PD) | 0 |  |
| Keur Lamine Diame | 13°37’N/16°15’W | 16-19/09/2002 | 22 (HD/PD) | 0 |  |
| Santhie Ram | 13°37’N/16°14’W | 20-22/09/2002 | 50 (HD/PD) | 0 |  |
| Tiolé (Dagana) | 16°29’N/15°00'W | 08/11/2007 | 10 (HD/PD) | 5 (50%) | *O. sonrai* ssp. |
| Mbouba (Podor) | 16°11'N/14°00'W | 10/11/2007 | 10 (F) | 10 (100%) | *O. sonrai* |
| Amadi Ounaré | 15°21’N/13°01'W | 11/11/2007 | 10 (F) | 4 (40%) | *O. sonrai* |
| Soulkhou Thissé | 14°03’N/15°31’W | 28-29/08/2008 | 30 (HD/PD) | 2 (6.6%) | *O. sonrai* |
| Maka Gouye | 13°48’N/14°56’W | 30-31/08/2008 | 24 (HD/PD) | 5 (21%) | *O. sonrai* |
| Diokoul 1 | 14°02’N/14°33’W | 01/09/2008 | 30 (HD/PD)* | 0* |  |
| Diokoul 2 | 14°01’N/14°33’W | 01/09/2008 |  |  |  |
| Koulor 1 | 13°58’N/13°24’W | 05-06/09/2008 | 30 (HD)* | 0* |  |
| Koulor 2 | 14°06’N/13°24’W | 05/09/2008 |  |  |  |
| Koulor 3 | 14°00’N/13°24’W | 05/09/2008 |  |  |  |
| Didé | 13°58’N/12°20’W | 06-07/09/2008 | 30 (HD) | 22 (73%) | *O. sonrai* |
| Dar Salam | 13°15’N/13°12’W | 12/11/2007 | 30 (HD/PD) | 0 |  |
| Saboya 1 | 13°37’N/16'°03’W | 14/09/2008 | 30 (HD/PD)* | 0* |  |
| Saboya 2 | 13°37’N/16'°04’W | 14/09/2008 |  |  |  |
| Silamé | 13°38’N/14°27’W | 02-03/09/2008 | 30 (HD/PD/N) | 0 |  |
| Douta Passi | 13°37’N/14°01W | 04/09/2008 | 30 (HD/PD) | 0 |  |
| Keur Ayip | 13°35’N/15°37’W | 01/03/2008 | 15 (N) | 12 (80%) | *O. sonrai* |
| Diana | 13°32’N/12°51’W | 09-10/09/2008 | 30 (HD) | 25 (83%) | *O. sonrai* |
| Sinthiou Djidé | 13°28’N/15°00’W | 03/03/2008 | 30 (HD/PD/N) | 0 |  |
| Saré Rawnadjidéré | 13°01’N/15°00’W | 04/03/2008 | 30 (HD/PD) | 0 |  |
| Mballocounda 1 | 13°00’N/14°01’W | 05/03/2008 | 30 (HD)* | 0* |  |
| Mballocounda 2 | 13°01’N/14°02’W | 05/03/2008 |  |  |  |
| Mballocounda 3 | 13°00’N/14°02’W | 05/03/2008 |  |  |  |
| Missira 1 | 13°31’N/13°30’W | 06/03/2008 | 30 (HD/PD)* | 0* |  |
| Missira 2 | 13°31’N/13°31’W | 06/03/2008 |  |  |  |
| Bokolako | 13°36’N/12°32’W | 10-11/09/2008 | 30 (HD) | 0 |  |
| Sadatou | 13°38’N/12°14’W | 10-11/09/2008 | 30 (HD/PD) | 0 |  |
| Djiro Mantoro 1 | 13°02’N/16°02’W | 08-09/03/2003 | 30 (N/F)* | 0* |  |
| Djiro Mantoro 2 | 13°01’N/16°02’W | 08-09/03/2003 |  |  |  |
| Abéné | 12°59’N/16°44’W | 10-11/03/2003 | 30 (N) | 0 |  |
| Tiel (Linguère) | 15°00’N/15°01’W | 13/03/2008 | 10 (N) | 8 (80%) | *O. sonrai* |
| Ranérou | 15°09’N/14°10’W | 13/03/2008 | 10 (N) | 5 (50%) | *O. sonrai* |
| Noto (Thiès) | 15°00’N/17°01’W | 14/03/2008 | 10 (N) | 3 (30%) | *O. sonrai* |
| Hann Centre IRD | 14°43’N/17°35’W | 17/10/2008 | 12 (PD) | 10 (83%) | *O. sonrai* |
| Hann Parc Zoologique | 14°43’N/17°35’W | 17/10/2008 | 18 (N) | 13 (72%) | *O. sonrai* |
| ***Total Senegal*** |  |  | **2, 861** | **474 (16.5%)** |  |
| **Mali** |  |  |  |  |  |
| Diougounté | 14°07’N/09°58’W | 13/12/2003 | 30 (HD/PD) | 11 (37%) | *O. sonrai* |
| Niamou 1 | 14°01’N/08°02’W | 14-15/12/2003 | 60 (HD/PD/N)* | 0* |  |
| Niamou 2 | 13°58’N/08°04’W | 15/12/2003 |  |  |  |
| Laminibougou 1 | 14°04’N/06°02’W | 17/12/2003 | 60 (HD/PD/N)* | 0* |  |
| Laminibougou 2 | 14°09’N/06°05’W | 15-16/12/2003 |  |  |  |
| Sofara / Ouo 1 | 14°00’N/04°00’W | 03/02/2004 | 60 (HD/N/F)* | 0* |  |
| Sofara / Ouo 2 | 14°03’N/03°51’W | 03/02/2004 |  |  |  |
| Sofara / Ouo 3 | 14°01’N/03°46’W | 03/02/2004 |  |  |  |
| Sofara / Ouo 4 | 14°09’N/04°06’W | 04/02/2004 |  |  |  |
| Tin-Bîdêne 1 | 19°01’N/01°50’E | 06-07/02/2004 | 60 (N)* | 0* |  |
| Tin-Bîdêne 2 | 19°00’N/01°46’E | 06-07/02/2004 |  |  |  |
| In-Tebezas | 18°01’N/01°49’E | 08-09/02/2004 | 60 (N)* | 0* |  |
| In-Tebezas | 17°56’N/01°46’E | 08-09/02/2004 |  |  |  |
| Massif Amastaouas 1 | 17°01’N/02°06’E | 10-12/02/2004 | 60 (N)* | 0* |  |
| Massif Amastaouas 2 | 17°02’N/02°09’E | 10-12/02/2004 |  |  |  |
| NW Ménaka | 16°05’N/02°09’E | 12-13/02/2004 | 60 (N) | 0 |  |
| Gaoudel 1 | 16°00’N/04°05’W | 19/01/2005 | 60 (N/F)* | 0* |  |
| Gaoudel 2 | 15°54’N/04°08’W | 18/01/2005 |  |  |  |
| Gaoudel 3 | 15°59’N/04°05’W | 18/01/2005 |  |  |  |
| Gaoudel 4 | 16°04’N/03°53’W | 19/01/2005 |  |  |  |
| Gaoudel 5 | 16°04’N/03°56’W | 19/01/2005 |  |  |  |
| Haoussa-Foulane 1 | 15°59’N/00°08’E | 22/01/2005 | 30 (N/F)* | 13 (43%)* | *O. sonrai* |
| Haoussa-Foulane 2 | 16°10’N/00°03’E | 22-23/01/2005 |  |  |  |
| Haoussa-Foulane 3 | 16°11’N/00°02’E | 23/01/2005 |  |  |  |
| Sama (Konna) | 14°55’N/03°53’W | 24/01/2005 | 10 (N/F) | 1 (10%) | *O. sonrai* |
| Molibana | 14°00’N/04°14’W | 25/01/2005 | 5 (N) | 2 (40%) | *O. sonrai* |
| San 1 | 13°18’N/04°51’W | 14-15/032005 | 30 (N/F)* | 0* |  |
| San 2 | 13°17’N/04°56'W | 14-15/032005 |  |  |  |
| Kénenko 1 | 13°05'N/07°15'W | 12-13/03/2005 | 30 (N/F)* | 0* |  |
| Kénenko 2 | 13°07'N/07°13'W | 12-13/03/2005 |  |  |  |
| Bamako Sud 1 | 12°30’N/08°08'W | 28-30/01/2005 | 60 (N/F)* | 0* |  |
| Bamako Sud2 | 12°33’N/08°07'W | 28-30/01/2005 |  |  |  |
| Makania | 14°04’N/11°09’W | 29/10/2005 | 15 (HD/PD) | 4 (27%) | *O. sonrai* |
| Guétala 1 | 13°59’N/09°33’W | 08/02/2007 | 33 (HD/PD/N)* | 0* |  |
| Guétala 2 | 13°59’N/09°35’W | 08/02/2007 |  |  |  |
| Guétala 3 | 13°56’N/09°32’W | 31/12/2006 |  |  |  |
| Kourougué 1 | 14°09’N/09°08’W | 30-31/12/2006 | 30 (HD/PD/N)* | 0* |  |
| Kourougué 2 | 14°10’'N/09°08’W | 30-31/12/2006 |  |  |  |
| Kourougué 3 | 14°11’N/09°08’W | 30-31/12/2006 |  |  |  |
| Kourougué 4 | 14°10’N/09°11’W | 30-31/12/2006 |  |  |  |
| Kourougué 5 | 14°11’N/09°09’W | 31/12/2006 |  |  |  |
| Sikoro | 14°00’N/07°01’W | 24-25/02/2005 | 30 (HD) | 0 |  |
| Sogoli | 13°58’N/05°28’W | 16-17/10/2007 | 30 (HD) | 28 (93%) | *O. sonrai* |
| Soumana | 13°54’N/04°58’W | 18/10/2007 | 30 (HD/PD/N) | 0 |  |
| Douagui (Bourem) 1 | 16°54’N/00°20’W | 25/01/2008 | 30 (HD/PD/N)* | 1 (3%)* | *O. sonrai* |
| Douagui (Bourem) 2 | 16°54’N/00°19’W | 25/01/2008 |  |  |  |
| Douagui (Bourem) 3 | 16°53’N/00°19’W | 25/01/2008 |  |  |  |
| Téméra | 16°59’N/00°57’W | 27/01/2008 | 15 (N) | 6 (40%) | *O. sonrai* |
| Gourma-Rharous 1 | 16°53’N/01°55’W | 07/10/2005 | 14 (N)* | 3 (21%)* | *O. sonrai* |
| Gourma-Rharous 2 | 16°53’N/01°56’W | 27/01/2008 |  |  |  |
| Wondoboumo | 16°40’N/02°58’W | 28/01/2008 | 12 (N) | 3 (25%) | *O. sonrai* |
| Yona 1 | 16°16’N/03°19’W | 29-30/01/2008 | 30 (N)* | 0* |  |
| Yona 2 | 16°16’N/03°20’W | 29-30/01/2008 |  |  |  |
| Niafounké 1 | 15°56’N/03°58’W | 31/01/2008 | 30 (N)* | 0* |  |
| Niafounké 2 | 15°52’N/04°00’W | 31/01/2008 |  |  |  |
| Brélingui 1 | 15°21’N/09°58’W | 27-29/09/2007 | 30 (HD/PD/N)* | 5 (17%)* | *O. sonrai* |
| Brélingui 2 | 15°22’N/09°55’W | 27-29/09/2007 |  |  |  |
| Brélingui 3 | 15°23’N/09°54’W | 27-29/09/2007 |  |  |  |
| Kouroukéré (Chkata) | 15°24’N/09°29’W | 29-30/09/2007 | 30 (HD/PD/N) | 22 (73%) | *O. sonrai* |
| Binéou-Niakaté | 15°27’N/08°54''W | 01/10/2007 | 30 (HD/PD) | 12 (40%) | *O. sonrai* |
| Mounta | 15°20’N/08°30’W | 02/10/2007 | 30 (HD/PD) | 12 (40%) | *O. sonrai* |
| Birou-Niakaté 1 | 15°27’N/08°04’W | 03/10/2007 | 30 (HD/PD)* | 0* |  |
| Birou-Niakaté 2 | 15°25’N/08°06’W | 03/10/2007 |  |  |  |
| Mantyonga | 15°20’N/07°27’W | 04/10/2007 | 30 (HD/PD) | 0 |  |
| Arhor | 15°24’N/06°55’W | 05-06/10/2007 | 12 (HD/PD) | 0 |  |
| Dar Es Salam 1 | 15°23’N/06°33’W | 07/10/2007 | 30 (PD/N)* | 0* |  |
| Dar Es Salam 2 | 15°22’N/06°33’W | 07/10/2007 |  |  |  |
| Dar Es Salam 3 | 15°24’N/06°32’W | 07/10/2007 |  |  |  |
| Bourgou-Siratigui | 15°23’N/05°28’W | 15/10/2007 | 30 (HD/PD/N) | 0 |  |
| Kounssoum 1 | 15°30’N/00°34’E | 22-23/06/2005 | 30 (N/F)* | 21 (70%)* | *O. sonrai* |
| Kounssoum 2 | 15°30’N/00°33’E | 22-23/06/2005 |  |  |  |
| Kounssoum 3 | 15°37’N/00°30’E | 24/06/2005 |  |  |  |
| Labbézanga 1 | 15°00’N/00°30’E | 24-26/06/2005 | 42 (N/F)* | 0* |  |
| Labbézanga 2 | 14°59’N/00°39’E | 24-26/06/2005 |  |  |  |
| Labbézanga 3 | 15°09’N/00°41’E | 26/06/2005 |  |  |  |
| Soukounala 1 | 15°01’N/10°58’W | 18/05/2006 | 30 (N/F/HD)* | 13 (43%)* | *O. sonrai* |
| Soukounala 2 | 15°02’N/10°58’W | 19/05/2006 |  |  |  |
| Monsombougou 1 | 15°00'N/10°00'W | 21/01/2006 | 30 (N/HD/PD)* | 22 (73%)* | *O. sonrai* |
| Monsombougou 2 | 14°58'N/10°01'W | 22/01/2006 |  |  |  |
| Lanbangoubo | 15°07'N/09°06'W | 23/01/2006 | 30 (HD/PD/N/F) | 19 (63%) | *O. sonrai* |
| Ziguim | 14°58'N/08°30’W | 16/02/2007 | 30 (HD/PD) | 0 |  |
| Bama 1 | 15°07’N/08°00’W | 25-26/01/2006 | 60 (HD/PD/N/F)* | 0* |  |
| Bama 2 | 15°08’N/08°01’W | 25-26/01/2006 |  |  |  |
| Bama 3 | 15°06’N/07°59’W | 25/01/2006 |  |  |  |
| Bama 4 | 15°07’N/08°01’W | 25-26/01/2006 |  |  |  |
| Bama 5 | 15°05’N/07°57’W | 26/01/2006 |  |  |  |
| Demba Sala | 14°59’N/07°31’W | 20/02/2007 | 30 (HD) | 27 (90%) | *O. sonrai* |
| Akor 1 | 15°03’N/07°08’W | 27-28/01/2006 | 60 (N/HD/PD)* | 0* |  |
| Akor 2 | 14°52’N/06°59’W | 27-28/01/2006 |  |  |  |
| Akor 3 | 14°52’N/06°58’W | 28/01/2006 |  |  |  |
| Akor 4 | 14°55’N/07°02’W | 29/01/2006 |  |  |  |
| Akor 5 | 14°54’N/07°01’W | 29/01/2006 |  |  |  |
| Driss 1 | 14°56’N/06°33’W | 08-9/10/2007 | 30 (N/HD/PD)* | 0* |  |
| Driss 2 | 14°55’N/06°31’W | 08-9/10/2007 |  |  |  |
| Kourmangoubé 1 | 14°54’N/06°03’W | 30/01/2006 | 60 (N/HD/PD)* | 0* |  |
| Kourmangoubé 2 | 14°53’N/06°07’W | 31/01/2006 |  |  |  |
| Toulé | 15°08'N/05°34’W | 13-14/10/2007 | 30 (HD/PD) | 0 |  |
| Dianguirdé 1 | 14°30’N/09°01’W | 06/02/2007 | 30 (HD/N)* | 15 (50%)* | *O. sonrai* |
| Dianguirdé 2 | 14°29’N/09°00’W | 07/02/2007 |  |  |  |
| Tonkasimi 1 | 14°30’N/08°31’W | 14/02/2007 | 30 (N/HD/PD)* | 16 (53.3%)* | *O. sonrai* |
| Tonkasimi 2 | 14°29’N/08°32’W | 15/02/2007 |  |  |  |
| Kolomina 1 | 14°29’N/08°01’W | 17/02/2007 | 30 (N/HD/PD)* | 15 (50%)* | *O. sonrai* |
| Kolomina 2 | 14°28’N/08°01’W | 18/02/2007 |  |  |  |
| Seymana | 14°30’N/07°31’W | 19/02/2007 | 30 (HD/PD) | 0 |  |
| Naouléna | 14°29’N/07°08’W | 23-24/02/2007 | 30 (HD) | 0 |  |
| Nyoko | 14°34’N/06°25’W | 09-10/10/2007 | 30 (HD/PD) | 0 |  |
| Kandyourou | 14°36’N/06°08'W | 11/10/2007 | 30 (HD) | 0 |  |
| Malémana | 14°33’N/05°25’W | 12-13/10/2007 | 30 (HD) | 0 |  |
| Piétaga 1 | 14°34’N/04°10’W | 02/02/2008 | 12 (N)* | 2 (17%)* | *O. sonrai* |
| Piétaga 2 | 14°31’N/04°11’W | 02/02/2008 |  |  |  |
| Djimékourou 1 | 13°30’N/11°00’W | 06/05/2006 | 60 (N/HD/PD)* | 0* |  |
| Djimékourou 2 | 13°32’N/11°01’W | 07/05/2006 |  |  |  |
| Kassama 1 | 13°03’N/11°06’W | 03/05/2006 | 60 (N/HD/PD)* | 0* |  |
| Kassama 2 | 13°03’N/11°07’W | 04/05/2006 |  |  |  |
| Kassama 3 | 13°03’N/11°10’W | 05/05/2006 |  |  |  |
| Billy 1 | 13°34’N/10°01’W | 09/02/2007 | 30 (HD/N)* | 0* |  |
| Billy 2 | 13°34’N/10°02’W | 10/02/2007 |  |  |  |
| Talikola 1 | 13°30’N/09°30’W | 11/02/2007 | 30 (N/HD/PD)* | 0* |  |
| Talikola 2 | 13°28’N/09°30’W | 12/02/2007 |  |  |  |
| Kolé 1 | 13°29’N/08°59’W | 13/02/2007 | 30 (N/HD/PD)* | 0* |  |
| Kolé 2 | 13°28’N/08°59’W | 13/02/2007 |  |  |  |
| Djinagué 1 | 12°59’N/09°52’W | 09/05/2006 | 60 (N/HD)* | 0* |  |
| Djinagué 2 | 13°00’N/09°52’W | 10/05/2006 |  |  |  |
| Yacoubabougou 1 | 13°00’N/08°55’W | 11/05/2006 | 60 (N/HD/PD)* | 0* |  |
| Yacoubabougou 2 | 13°00’N/08°54’W | 12/05/2006 |  |  |  |
| Yacoubabougou 3 | 13°01’N/08°53’W | 1305/2006 |  |  |  |
| Gouakoulou | 13°30’N/08°03’W | 16-17/05/2006 | 60 (N/HD) | 0 |  |
| Kodian 1 | 13°03’N/07°56’W | 14/05/2006 | 60 (F/N/HD)* | 0* |  |
| Kodian 2 | 13°04’N/07°57’W | 14/05/2006 |  |  |  |
| Kodian 3 | 13°02’N/07°57’W | 15/05/2006 |  |  |  |
| Kodian 4 | 13°03’N/07°57’W | 15/05/2006 |  |  |  |
| Markala 1 | 13°42’N/06°04’W | 01/02//2006 | 8 (N)* | 0* |  |
| Markala 2 | 13°42’N/06°04’W | 25/02//2007 |  |  |  |
| Dona | 13°12’N/05°54’W | 04/02/2008 | 30 (N) | 0 |  |
| Wesso | 13°27’N/04°49’W | 20/10/2007 | 30 (HD) | 0 |  |
| ***Total Mali*** |  |  | **2,458** | **273 (11.1%)** |  |
| **Guinea** |  |  |  |  |  |
| Dima (Pont Gambie) 1 | 12°00’N/11°49’W | 14/02/2006 | 60 (HD/N/F)* | 0* |  |
| Dima (Pont Gambie) 2 | 12°01’N/11°49’W | 14-15/02/2006 |  |  |  |
| Dima (Pont Gambie) 3 | 12°02’N/11°49’W | 15/02/2006 |  |  |  |
| Dima (Pont Gambie) 4 | 11°57’N/11°50’W | 16/02/2006 |  |  |  |
| Siréya 1 | 11°02’N/11°56’W | 17/02/2006 | 60 (HD/PD)* | 0* |  |
| Siréya 2 | 11°02’N/11°58’W | 18/02/2006 |  |  |  |
| Kindia | 10°05’N/12°50’W | 26/04/2008 | 30 (N) | 0 |  |
| Kalankalan | 10°06’N/08°53’W | 18/04/2008 | 30 (HD/PD) | 0 |  |
| Oueye | 08°02'N/08°56’W | 20/04/2008 | 30 (HD/PD) | 0 |  |
| Yatia | 10°01’N/10°58’W | 28/10/2012 | 30 (HD/PD) | 0 |  |
| Kunda | 10°50’N/13°48’W | 01/11/2012 | 30 (HD/PD) | 0 |  |
| ***Total Guinea*** |  |  | **270** | **0 (0%)** |  |
| **Togo** |  |  |  |  |  |
| Konkoré 1 | 10°33’N/00°12’E | 07/01/2008 | 30 (N/HD)* | 0* |  |
| Konkoré 2 | 10°44’N/00°11’E | 08/01/2008 |  |  |  |
| Alédjo 1 | 09°15’N/01°13’E | 09/01/2008 | 30 (HD/PD)* | 0* |  |
| Alédjo 2 | 09°15’N/01°13’E | 10/01/2008 |  |  |  |
| Diguengué | 08°05’N/00°38’E | 11-12/01/2008 | 30 (HD/PD) | 0 |  |
| Sodo-Zion | 07°19’N/00°50’E | 13/01/2008 | 30(HD/PD) | 0 |  |
| Sagonou | 06°16’N/01°18’E | 15/01/2008 | 30 (HD/PD) | 0 |  |
| ***Total Togo*** |  |  | **150** | **0 (0%)** |  |
| **Chad** |  |  |  |  |  |
| Dile Tchiloumdom | 14°01’N/14°09’E | 19/02/2003 | 60 (N) | **0%** |  |
| Méchiméré | 13°49’N/15°54’E | 15-17/01/2003 | 60 (HD/F/N) | **0%** |  |
| Djedaa | 14°01’N/18°01’E | 23-24/01/2003 | 60 (N) | 0 |  |
| Al Miamine | 13°46’N/19°44’E | 21-22/01/2003 | 60 (N/F) | 0 |  |
| Sounoute (Saguéré) | 14°09’N/21°50’E | 19-20/01/2003 | 60 (HD/F) | 0 |  |
| Doué 1 | 09°15’N/14°42’E | 10/02/2003 | 60 (HD/F)* | 0* |  |
| Doué 2 | 09°16’N/14°41’E | 08-09/02/2003 |  |  |  |
| Mataya | 11°59’N/18°02’E | 25/01/2003 | 35 (N/HD)* | 0* |  |
| Ati | 13°00’N/18°06’E | 24/01/2003 |  |  |  |
| Ndjaména | 12°01’N/15°06’E | 17/02/2003 | 63 (F/HD)* | 0* |  |
| Toukra-Arabe | 12°05’N/15°06’E | 16/02/2003 |  |  |  |
| ***Total Chad*** |  |  | **458** | **0 (0%)** |  |
| **Cameroon** |  |  |  |  |  |
| Yik 1 | 12°30’N/14°35’E | 05-07/02/2003 | 60 (HD/F)* | **0*** |  |
| Yik 2 | 12°25’N/14°52’E | 06/02/2003 |  |  |  |
| Yik 3 | 12°30’N/14°36’E | 05-07/02/2003 |  |  |  |
| Kossa 1 | 11°07’N/14°19’E | 02/02//2003 | 79 (HD/F/N)* | 0* |  |
| Kossa 2 | 11°09’N/14°22’E | 01/02//2003 |  |  |  |
| Kossa 3 | 11°05’N/14°21’E | 03/02//2003 |  |  |  |
| Mogom 1 | 10°30’N/14°25’E | 29-30/01/2003 | 79 (HD/F/N)* | 0* |  |
| Mogom 2 | 10°20’N/14°27’E | 30/01/2003 |  |  |  |
| Diéra | 08°31’N/13°30’E | 12/02/2003 | 60 (HD/N/F) | 0 |  |
| ***Total Cameroon*** |  |  | **278** | **0 (0%)** |  |
| **Niger** |  |  |  |  |  |
| Simiri 1 | 14°08’N/02°08’E | 11-12/01/2004 | 60 (HD/N/F)* | 0* |  |
| Simiri 2 | 14°02’N/02°05’E | 11-12/01/2004 |  |  |  |
| Kolifo 1 | 13°59’N/04°00’E | 24-25/01/2004 | 60 (N/F/HD)* | 0* |  |
| Kolifo 2 | 13°58’N/03°56’E | 25/01/2004 |  |  |  |
| Tékhé | 14°01’N/06°01’E | 13-14/01/2004 | 60 (HD/PD) | 0 |  |
| Mazadaoua 1 | 14°00’N/07°59’E | 16-17/01/2004 | 60 (HD/F/N)* | 0* |  |
| Mazadaoua 2 | 14°00’N/08°00’E | 16-17/01/2004 |  |  |  |
| Gidyo 1 | 14°00’N/10°02’E | 18-19/01/2004 | 60 (HD/N/PD)* | 0* |  |
| Gidyo 2 | 13°59’N/10°02’E | 19/01/2004 |  |  |  |
| Puits artésien de Mir | 14°00’N/12°00’E | 21-22/01/2004 | 60 (HD/PD/N) | 0 |  |
| Tiloa | 15°09’N/02°04’E | 14-15/02/2004 | 60 (N/F) | 0 |  |
| Piliki | 13°08’N/01°57’E | 17-18/02/2004 | 60 (HD/PD) | 0 |  |
| Farié 1 | 14°01’N/01°32’E | 23-24/02/2005 | 30 (N, F)* | 0* |  |
| Farié 2 | 14°07’N/01°31’E | 23-24/02/2005 |  |  |  |
| Ayorou | 14°42’N/00°55’E | 23/01/2008 | 30 (N) | 0 |  |
| Niamey 1 | 13°32’N/02°01’E | 24-25/02/2005 | 30 (N/F)* | 0* |  |
| Niamey 2 | 13°28’N/02°08’E | 24-25/02/2005 |  |  |  |
| Tchintoulous 1 | 18°35’N/08°46’E | 01/03/2005 | 30 (N)* | 0* |  |
| Tchintoulous 2 | 18°34’N/08°47’E | 01/03/2005 |  |  |  |
| Taggout | 18°43’N/08°30’E | 01/03/2005 |  |  |  |
| Tchi-n-Taghoda | 18°54’N/08°33’E | 01/03/2005 |  |  |  |
| Tessa-n-Jakanar | 18°38’N/09°05’E | 02/03/2005 |  |  |  |
| Iférouane | 19°04’N/08°26’E | 28/02/2005 | 31 (N)* | 0* |  |
| Taghmert | 19°06’N/09°02’E | 03/03/2005 |  |  |  |
| Adrar Tamgak | 19°09’N/08°56’E | 03/03/2005 |  |  |  |
| Adrar Chiriet | 19°17’N/09°14’E | 04/03/2005 |  |  |  |
| ***Total Niger*** |  |  | **631** | **0 (0%)** |  |
| **Benin** |  |  |  |  |  |
| Boutéré | 10°51’N/02°08’E | 20-21/02/2004 | 60 (HD/PD/N) | 0% |  |
| Kpêré 1 | 09°59’N/01°53’E | 22-23/02/2004 | 60 (HD/PD/N)* | 0* |  |
| Kpêré 2 | 10°00’N/01°54’E | 23/02/2004 |  |  |  |
| Igbéré 1 | 08°59’N/01°57’E | 24-25/02/2004 | 60 (HD/PD/N)* | 0* |  |
| Igbéré 2 | 09°00’N/01°59’E | 24/02/2004 |  |  |  |
| Doyissa 1 | 07°59’N/01°59’E | 26-27/02/2004 | 60 (HD/F/N)* | 0* |  |
| Doyissa 2 | 07°59’N/01°58’E | 27/02/2004 |  |  |  |
| Lanta | 07°06’N/01°52’E | 28/02/2004 | 60 (N/F) | 0 |  |
| ***Total Benin*** |  |  | **300** | **0 (0%)** |  |
| **Gambia** |  |  |  |  |  |
| Lansar | 13°35’N/15°37’W | 02/03/2008 | 24 (HD/PD) | 14 (58%) | *O. sonrai* |
| Joben | 13°46’N/14°58’W | 31/08/2008 | 6 (PD) | 1 (17%) | *O. sonrai* |
| ***Total Gambia*** |  |  | **30** | **15 (50%)** |  |
| **Guinea Bissau** |  |  |  |  |  |
| Erkunda | 12°01’N/14°57’W | 08/02/2011 | 30 (N) | **0** |  |
| ***Total Guinea Bissau*** |  |  | **30** | **0 (0%)** |  |
| **Liberia** |  |  |  |  |  |
| Bopolu | 07°04’N/10°29’W | 25/03/2009 | 30 (HD/PD) | 0% |  |
| Zwedru | 05°35’N/08°32’W | 28/03/2009 | 30 (HD/PD) | 0% |  |
| ***Total Liberia*** |  |  | **60** | **0 (0%)** |  |
| **Spain** |  |  |  |  |  |
| Torremolinos | 36°38’N/04°30’W | 18/07/2010 | 7 (N) | 2 (29%) | *O. occidentalis* |
| Colmenar | 36°55’N/04°19’W | 18/07/2010 | 10 (F) | 0 | *O. occidentalis* |
| ***Total Spain*** |  |  | **17** | **2 (11.7%)** |  |
| **Ivory Coast** |  |  |  |  |  |
| Pakobo 1 | 06°07’N/04°55’W | 18/10/2012 | 30 (HD/PD)* | **0** |  |
| Pakobo 2 | 06°05’N/04°55’W | 18/10/2012 |  |  |  |
| Pakobo 3 | 06°12’N/04°57’W | 18/10/2012 |  |  |  |
| Allakro | 07°56’N/05°03’W | 19/10/2012 | 30 (HD) | 0 |  |
| Detikaha | 09°55’N/05°10’W | 20/10/2012 | 30 (HD/PD) | 0 |  |
| ***Total Ivory Coast*** |  |  | **90** | **0%** |  |
| **Burkina Faso** |  |  |  |  |  |
| Toulfé 1 | 13°56’N/02°00’W | 31/01-01/02/2004 | 60 (N/HD)* | **0*** |  |
| Toulfé 2 | 13°53’N/01°57’W | 01/02/2004 |  |  |  |
| Ndjiongba 1 | 14°00’N/00°00’W | 29-30/01/2004 | 60 (N)* | 0* |  |
| Ndjiongba 2 | 13°54’N/00°05’W | 29-30/01/2004 |  |  |  |
| Ndjiongba 3 | 14°04’N/00°02’W | 30/01/2004 |  |  |  |
| Mangou | 12°04’N/01°52’E | 27-28/01/2004 | 60 (HD/PD/N) | 0 |  |
| Noumoukina | 11°50’N/04°52’W | 21/10/2012 | 30 (HD) | 0 |  |
| ***Total Burkina Faso*** |  |  | **210** | **0 (0%)** |  |
|  |  |  |  |  |  |
| **TOTAL** |  |  | **9,858** | **1,224 (12.4%)** |  |

*: Compiled site stations data

F: Fields

N: Natural habitat

PD: Peridomestic

HD: Human dwellings
