# Supplementary material for: The Epidemiology and Geographic Distribution of Relapsing Fever Borreliosis in West and North Africa, with a Review of the Ornithodoros erraticus Complex (Acari: Ixodida)
Source: PLoS One. 2013 Nov 4;8(11):e78473. doi: 10.1371/journal.pone.0078473 (PMC3817255; doi:10.1371/journal.pone.0078473)
Supplement: Table S2 — Detailed results of small mammals surveys. (DOCX) [file pone.0078473.s002.docx]

| **Supplementary Table S2.** Detailed results of small mammals surveys* | | | | | | | | | | |
| --- | --- | --- | --- | --- | --- | --- | --- | --- | --- | --- |
| **Study area** | **Coordinate of**  **sampling sites** | **Date** | **No. of**  **trap-nights** | **Habitat**  **sampled** | | **Mammals species (No, I/T)**** | | | | |
|  |  |  |  |  | |  | | |  | |
| **Senegal** | |  |  |  | |  | | |  | |
| Dielmo | 13°43'N/16°24'W | 17-24/04/2002 | 990 | HD | | *Mastomys erythroleucus* (30, 6/23) | | | | |
|  |  |  |  |  | | *Crocidura olivieri* (13, 8/11) | | | | |
|  |  |  |  |  | | *Mus musculus* (10, 1/7) | | | | |
|  |  |  |  |  | | *Arvicanthis niloticus* (5, 0/5) | | | | |
|  |  |  |  |  | | *Rattus rattus* (3, 2/3) | | | | |
|  |  |  |  |  | | *Cricetomys gambianus* (2, 0/1) | | | | |
|  |  |  |  |  | | *Praomys daltoni* (2, 0/2) | | | | |
| Ndiop | 13°41'N/16°22'W | 6-13/03/2002 | 857 | HD | | *Crocidura olivieri* (28, 1/22) | | | | |
|  |  |  |  |  | | *Mastomys erythroleucus* (25, 2/15) | | | | |
|  |  |  |  |  | | *Mus musculus* (4, 0/0) | | | | |
|  |  |  |  |  | | *Rattus rattus* (3, 0/2) | | | | |
|  |  |  |  |  | | *Arvicanthis niloticus* (1, 1/1) | | | | |
| ***Total Senegal*** |  |  | **1,847** |  | | **Total specimens*:* 126, 21/92** | | | | |
| **Mauritania** | |  |  |  | |  | | |  | |
| Soufa | 15°56'N/12°0 l'W | 13/11/2003 | 568 | N, HD | | *Gerbillus nanus* (3, 0/3) | | | | |
|  |  |  |  |  | | *Taterillus* sp. (1, 0/1) | | | | |
|  |  |  |  |  | | *Mastomys erythroleucus* (6, 0/6) | | | | |
|  |  |  |  |  | | *Atelerix albiventris* (1, 0/1) | | | | |
|  |  |  |  |  | | *Desmodilliscus braueri* (2, 0/0) | | | | |
| Nbeika | 17°59'N/12°14'W | 18-19/11/2003 | 277 | N, HD | | *Gerbillus* sp. (1, 0/0) | | | | |
|  |  |  |  |  | | *Gerbillus nanus* (1, 0/1) | | | | |
|  |  |  |  |  | | *Gerbillus campestris* (4, 0/4) | | | | |
|  |  |  |  |  | | *Jaculus jaculus* (21, 0/21) | | | | |
|  |  |  |  |  | | *Gerbillus gerbillus* (1, 0/1) | | | | |
|  |  |  |  |  | | *Gerbillus tarabuli (*1, 0/1) | | | | |
| Rachid | 18°47'N/11° 41 'W | 20-21/11/2003 | 262 | N, HD | | *Acomys airensis* (34, 0/24) | | | | |
|  |  |  |  |  | | *Gerbillus* sp. (3, 0/2) | | | | |
|  |  |  |  |  | | *Gerbillus campestris* (2, 0/2) | | | | |
|  |  |  |  |  | | *Gerbillus henleyi* (1, 0/1) | | | | |
| Oujeft | 20°00'N/13°03'W | 21/11/2003 | 247 | N, HD | | *Gerbillus tarabuli* (17, 0/12) | | | | |
|  |  |  |  |  | | *Gerbillus nanus* (2, 0/2) | | | | |
|  |  |  |  |  | | *Gerbillus campestris* (1, 0/1) | | | | |
|  |  |  |  |  | | *Acomys airensis* (1, 0/1) | | | | |
|  |  |  |  |  | | *Mus musculus* (9, 0/8) | | | | |
|  |  |  |  |  | | *Felovia vae* (1, 0/1) | | | | |
| Touajil | 22°07'N/12°41'W | 29/1112003 | 191 | F | | *Gerbillus gerbillu*s (10, 0/7) | | | | |
|  |  |  |  |  | | *Gerbillus tarabuli* (1, 0/1) | | | | |
| Guelb el Dlim | 22°59'N/12°00'W | 30/11/2003 | 163 | N | | *Gerbillus* sp. (2, 0/2) | | | | |
|  |  |  |  |  | | *Jaculus jaculus* (2, 0/2) | | | | |
| Aïn El Taya | 20°16'N/13°07'W | 20-24/10/2006 | 549 | N | | *Acomys chudeaui* (17, 0/14) | | | | |
|  |  |  |  |  | | *Jaculus jaculus*. (2, 0/2) | | | | |
|  |  |  |  |  | | *Gerbillus tarabuli* (4,0/3) | | | | |
|  |  |  |  |  | | *lctonyx striatus* (1, 0/1) | | | | |
|  |  |  |  |  | | *Gerbillus pyramidum* (3, 0/3) | | | | |
|  |  |  |  |  | | *Gerbillus campestris* (2, 0/2) | | | | |
|  |  |  |  |  | | *Gerbillus gerbillus* (1, 0/1) | | | | |
|  |  |  | Manual catches |  | | *Gerbillus* sp (21, 0/0) | | | | |
| Tiguent | 17°12'N/16°04'W | 28-30/10/2006 | 280 | N | | *Gerbillus gerbillus* (2, 0/2) | | | | |
|  |  |  |  |  | | *Gerbillus tarabuli* (35, 0/26) | | | | |
| Akjoujt | 19°44'N/14°22'W | 17-20/10/2006 | 518 | N, F | | *Gerbillus nanus* (1, 0/0) | | | | |
|  |  |  |  |  | | *Gerbillus tarabuli* (1, 0/1) | | | | |
|  |  |  |  |  | | *Gerbillus pyramidum* (15, 0/15) | | | | |
|  |  |  |  |  | | *Gerbillus* sp. (1, 0/1) | | | | |
|  |  |  |  |  | | *Hemiechinus aethiopicus* (1, 0/1) | | | | |
|  |  |  |  |  | | *Jaculus jaculus* (1, 0/0) | | | | |
|  |  |  |  |  | | *Pachyuromys duprasi* (1, 0/0) | | | | |
| Oumou Tunsi | 18°43'N/15°36'W | 24-25/10/2006 | 140 | N | | *Gerbillus* sp. (2, 0/2) | | | | |
|  |  |  |  |  | | *Gerbillus nanus* (1, 0/1) | | | | |
|  |  |  |  |  | | *Hemiechinus aethiopicus* (1, 0/1) | | | | |
| Lemcid | 18°39'N/16°06'W | 25-27/10/2006 | 280 | N | | *Gerbillus tarabuli* (6, 2/5) | | | | |
|  |  |  |  |  | | *Gerbillus gerbillus* (17, 1/15) | | | | |
|  |  |  |  |  | | *Gerbillus nanus* (2, 0/1) | | | | |
| South of Nouakchott | 17°23'N/16°03'W | 27-28/10/2006 | 140 | N | | *Xerus erythropus* (1, 0/0) | | | | |
|  |  |  |  |  | | *Gerbillus* sp. (1, 0/1) | | | | |
|  |  |  |  |  | | *Gerbillus tarabuli* (5, 0/4) | | | | |
| Keur Massène | 16°34'N/16°I7'W | 30/10-1/11/2006 | 225 | N | | *Xerus erythropus* (1, 0/1) | | | | |
|  |  |  |  |  | | *Taterillus arenarius* (7, 0/5) | | | | |
|  |  |  |  |  | | *Gerbillus gerbillus* (1, 0/1), | | | | |
| ***Total Mauritania*** |  |  | **5,357** |  | | **Total specimens : 282, 3/214** | | | | |
| **Mali** | |  |  |  | |  | | |  | |
| Diougounté | 14°07'N/09°58'W | 13/12/2003 |  | HD | | *Mastomys erythroleucus* (1, 1/1) | | | | |
| Tin-Bidêne | 19°01 'N/01°50'E | 5-8/02/2004 | 368 | N | | *Gerbillus tarabuli* (19, 0/19) | | | | |
|  |  |  |  |  | | *Gerbillus nanus* (7, 0/7) | | | | |
|  |  |  |  |  | | *Gerbillus pyramidum* (7, 0/7) | | | | |
|  |  |  |  |  | | *Gerbillus campestris* (1, 0/1) | | | | |
|  |  |  |  |  | | *Arvicanthis niloticus* (1, 0/1) | | | | |
|  |  |  |  |  | | *Jaculus jaculus* (3, 0/3) | | | | |
| ln- Tebezas | 18°01'N/01°49'E | 8-10/02/2004 | 175 | N | | *Gerbillus tarabul*i (5, 0/5) | | | | |
|  |  |  |  |  | | *Gerbillus nanu*s (2, 0/2) | | | | |
|  |  |  |  |  | | *Gerbillus pyramidum* (3, 0/3) | | | | |
|  |  |  |  |  | | *Arvicanthis niloticus* (1, 0/1) | | | | |
|  |  |  |  |  | | *Jaculus jaculus* (2, 0/2) | | | | |
| Massif Amastaouas | 17°0 l 'N/02°06'E | 10-12/02/2004 | 219 | N | | *Arvicanthis niloticus* (7, 0/7) | | | | |
|  |  |  |  |  | | *Gerbillus nanus* (5, 0/4) | | | | |
|  |  |  |  |  | | *Taterillus* *petteri* (2, 0/2) | | | | |
|  |  |  |  |  | | *Gerbillus tarabuli* (2, 0/2) | | | | |
|  |  |  |  |  | | *Jaculus jaculus* (6, 0/6) | | | | |
| Batamani-Daga | 14°52'N/04°03'W | 20-21/10/2007 | 200 | N | | *Mastomys huberti* (76, 1/76) | | | | |
|  |  |  |  |  | | *Mastomys natalensis* (12, 0/12) | | | | |
|  |  |  |  |  | | *Mastomys erythroleucus* (3, 0/3) | | | | |
|  |  |  |  |  | | *Arvicanthis* *ansorgei* (3, 0/3) | | | | |
|  |  |  |  |  | | *Arvicanthis niloticus* (7, 0/7) | | | | |
|  |  |  |  |  | | *Crocidura* *viaria* (9, 0/9) | | | | |
| Niamou | 14°0 l 'N/08°02'W | 26-27/10/2007 | 109 | HD | | *Mastomys natalensis* (36, 0/31) | | | | |
|  |  |  |  |  | | *Praomys daltoni* (1, 0/1) | | | | |
| Médine | 14°22'N/11°21 'W | 9-10/10/2007 | 195 | N, F | | *Taterillus* sp. (2, 0/2) | | | | |
|  |  |  |  |  | | *Gerbilliscus gambianus* (5, 0/4) | | | | |
|  |  |  |  |  | | *Mastomys erythroleucus* (1, 0/1) | | | | |
| Kolomina | 14°28'N/08°0I'W | 22-23/10/2007 | 110 | HD | | *Mastomys natalensis* (20, 6/19) | | | | |
|  |  |  |  |  | | *Praomys daltoni* (3, 1/3) | | | | |
| Argueta | 14°57'N/11°02'W | 11/10/2007 | 1 | HD | | *Praomys daltoni* (2, 0/2) | | | | |
| Topokoné | 15°02N/10°34'W | 13-14/10/2007 | 80 | F, HD | | *Praomys daltoni* (23, 3/22) | | | | |
| Monsombougou | 14°58'N/l0°0I'W | 15-17/10/2007 | 214 | N, HD | | *Taterillus* sp. (1, 0/1) | | | | |
|  |  |  |  |  | | *Praomys daltoni* (41, 6/41) | | | | |
|  |  |  |  |  | | *Arvicanthis nilolicus* (1, 0/1) | | | | |
|  |  |  |  |  | | *Crocidura* sp. (1, 0/1) | | | | |
| Hassilbarké-Maure | 14°54'N/09°24'W | 18-19/10/2007 | 141 | N, HD | | *Atelerix albiventris* (2, 0/1) | | | | |
|  |  |  |  |  | | *Gerbillus nigeriae* (l, 0/1 ) | | | | |
|  |  |  |  |  | | *Praomys daltoni* (57, 1/54) | | | | |
|  |  |  |  |  | | *Arvicanthis niloticus* (2, 0/2) | | | | |
|  |  |  |  |  | | *Desmodilliscus braueri* (1, 0/1) | | | | |
|  |  |  |  |  | | *Mastomys natalensis* (1, 1/1) | | | | |
| Sambé | 15°07'N/07°57'W | 24-25/10/2007 | 135 | N, HD, F | | *Taterillus* sp. (3, 0/3) | | | | |
|  |  |  |  |  | | *Xerus erythropus* (1, 0/1) | | | | |
|  |  |  |  | |  | | *Praomys daltoni* (44, 0/43) | | |  |
| ***Total Mali*** |  |  | **1,747** | |  | | **Total specimens: 433, 20/419** | | |  |
| **Niger** | |  |  | |  | |  |  | |  |
| Tiloa | 15°09'N/02°04'E | 13-15/02/2004 | 186 | | F, N | | *Gerbillus nigeriae* (21, 0/20) | | |  |
|  |  |  |  | |  | | *Acomys airensis* (3, 0/3) | | |  |
|  |  |  |  | |  | | *Gerbillus tarabuli* (11, 0/11) | | |  |
|  |  |  |  | |  | | *Arvicanthis niloticus* (1, 0/1) | | |  |
|  |  |  |  | |  | | *Mastomys erythroleucus* (6, 0/5) | | |  |
|  |  |  |  | |  | | *Hemiechinus aethiopicus* (1, 0/0) | | |  |
| Tékhé | 14°01'N/06°01'E | 13-16/01/2004 | Manual catches | | N | | *Gerbillus nigeriae* (2, 0/2) | | |  |
|  |  |  |  | |  | | *Desmodilliscus braueri* (1, 0/0) | | |  |
| Mazadaoua | 14°00'N/07°59'E |  | Manual catches | | NH | | *Gerbillus nigeriae* (4, 0/3) | | |  |
|  |  |  |  | |  | | *Gerbillus nanus* (1, 0/0) | | |  |
| Piliki | 13°08'N/01°57'E | 17-18/02/2004 | 203 | | HD, N | | *Arvicanthis* sp. (6, 0/0) | | |  |
|  |  |  |  | |  | | *Mastomys natalensis* (31, 0/28) | | |  |
|  |  |  |  | |  | | *M. erythroleucus* (35, 0/32) | | |  |
|  |  |  |  | |  | | *Gerbilliscu*s sp. (7, 0/4) | | |  |
|  |  |  |  | |  | | *Taterillus* *gracilis* (8, 0/6) | | |  |
| ***Total Niger*** |  |  | **389** | |  | | **Total specimens: 138, 0/115** | | |  |
| **Benin** | |  |  | |  | |  |  | |  |
| Boutéré | 10°51'N/02°08'E | 20-22/02/2004 | 428 | | HD, N | | *Gerbilliscus kempi* (1, 0/0) | | |  |
|  |  |  |  | |  | | *Gerbilliscus* sp. (4, 0/2) | | |  |
|  |  |  |  | |  | | *Mastomys erythroleucus* (8, 0/6) | | |  |
|  |  |  |  | |  | | *Lemniscomys* sp. (1, 0/1) | | |  |
|  |  |  |  | |  | | *Praomys daltoni* (1, 0/1) | | |  |
|  |  |  |  | |  | | *Heliosciurus gambianus* (1, 0/1) | | |  |
|  |  |  |  | |  | | *Atelerix albiventris* (1, 0/0) | | |  |
|  |  |  |  | |  | | *Taterillus* sp. (1, 0/1) | | |  |
|  |  |  |  | |  | | *Mastomys natalensis* (38, 0/20) | | |  |
| Igbéré | 08°59'N/01°57'E | 24-25/02/2004 | 243 | | N, HD | | *M. erythroleucus* (20, 0/18) | | |  |
|  |  |  |  | |  | | *Mastomys* sp. (13, 0/9) | | |  |
|  |  |  |  | |  | | *Lemniscomys bellieri* (2, 0/0) | | |  |
|  |  |  |  | |  | | *Lemniscomys* sp. (13, 0/13) | | |  |
|  |  |  |  | |  | | *Uranomys ruddi* (12, 0/12) | | |  |
|  |  |  |  | |  | | *Crocidura* sp. (2, 0/2) | | |  |
|  |  |  |  | |  | | *Gerbilliscus kempi* (2, 0/0) | | |  |
|  |  |  |  | |  | | *Gerbilliscus* sp. (5, 0/4) | | |  |
|  |  |  |  | |  | | *Praomys daltoni* (6, 0/6) | | |  |
|  |  |  |  | |  | | *Mastomys natalensis* (22, 0/10) | | |  |
|  |  |  |  | |  | | *Praomys derooi* (9, 0/8) | | |  |
|  |  |  |  | |  | | *Arvicanthis* sp. (1, 0/0) | | |  |
|  |  |  |  | |  | |  | | |  |
| Doyissa | 07°59'N/01°59'E | 26-27/02/2004 | 206 | | N, HD | | *Mastomys* sp. (6, 0/5) | | |  |
|  |  |  |  | |  | | *Crocidura* sp. (2, 0/2) | | |  |
|  |  |  |  | |  | | *M. erythroleucus* (17, 0/16) | | |  |
|  |  |  |  | |  | | *Uranomys ruddi* (2, 0/2) | | |  |
|  |  |  |  | |  | | *Mastomys natalensis* (23, 0/10) | | |  |
|  |  |  |  | |  | | *Praomys derooi* (6, 0/4) | | |  |
|  |  |  |  | |  | | *Rattus rattus* (4, 0/3) | | |  |
| Lanta | 07°06'N/01°52'E | 29/02/2004 | Manual catches | | N, HD | | *Mastomys natalensis* (9, 0/7) | | |  |
|  |  |  |  | |  | | *Rattus rattus* (7, 0/6) | | |  |
|  |  |  |  | |  | | *Cricetomys gambianus* (5, 0/3) | | |  |
|  |  |  |  | |  | | *Arvicathis rufinus* (5, 0/0) | | |  |
|  | |  |  | |  | | *Gerbilliscus kempi* (1, 0/0) | | |  |
| ***Total Benin*** | |  | **877** | |  | | **Total specimens: 249, 0/172** | | |  |
| **Chad** | |  |  | |  | |  | | |  |
| Sounoute (Saguéré) | 14°09'N/21°50'E | 19-20/01/2003 | 100 | | N | | *Atelerix albiventris* (1, 0/0) | | |  |
| Doué | 09°16'N/14°41'E | 8-10/02/2003 | 338 | | N, HD | | *Mastomys natalensis* (2, 0/2) | | |  |
|  |  |  |  | |  | | *Crocidura* sp. (1, 0/1) | | |  |
|  |  |  |  | |  | | *M. erythroleucus* (29, 0/29) | | |  |
|  |  |  |  | |  | | *M. kollmannspergeri* (16, 0/16) | | |  |
|  |  |  |  | |  | | *Gerbilliscus* sp. (1, 0/1) | | |  |
|  |  |  |  | |  | | *Uranomys ruddi* (1, 0/0) | | |  |
| Mataya | 11°59'N/18°02'E | 26/01/2003 | Manual catches | | HD | | *Mastomys natalensis* (3, 0/3) | | |  |
| ***Total Chad*** |  |  | **438** | |  | | **Total specimens: 54, 0/52** | | |  |
| **Cameroon** |  |  |  | |  | |  | | |  |
| Yik | 12°30'N/14°35'E | 4-6/02/2003 | 263 | | HD, N | | *Crocidura* sp. (4, 0/4) | | |  |
|  |  |  |  | |  | | *Mastomys natalensis* (3, 0/3) | | |  |
|  |  |  |  | |  | | *Gerbilliscus* sp. (13, 0/12) | | |  |
|  |  |  |  | |  | | *M. kollmannspergeri* (4, 0/4) | | |  |
|  |  |  |  | |  | | *Mastomys* sp. (7, 0/4) | | |  |
|  |  |  |  | |  | | *Taterillus lacustris* (7, 0/5) | | |  |
|  |  |  |  | |  | | *Atelerix albiventris* (2, 0/0) | | |  |
|  |  |  | Manual catches | | N | | *Desmodilliscus braueri* (5, 0/5) | | |  |
| Kossa | 11°07'N/14°19'E | 1-3/02/2003 | 338 | | HD, N | | *Arvicanthis* sp. (6, 0/4) | | |  |
|  |  |  |  | |  | | *Mastomys erythroleucus* (8, 0/8) | | |  |
|  |  |  |  | |  | | *Mastomys natalensis* (3, 0/3) | | |  |
|  |  |  |  | |  | | *Arvicanthis* sp. (21, 0/19) | | |  |
|  |  |  |  | |  | | *Gerbilliscus* sp. (8, 0/7) | | |  |
|  |  |  |  | |  | | *Mastomys erythroleucus* (2, 0/2) | | |  |
|  |  |  |  | |  | | *Lemniscomys zebra* (3, 0/3) | | |  |
|  |  |  |  | |  | | *Xerus erythropus* (3, 0/1) | | |  |
|  |  |  |  | |  | | *Gerbilliscus* sp. (16, 0/15) | | |  |
|  |  |  |  | |  | | *Taterillus* sp. (5, 0/2) | | |  |
|  |  |  |  | |  | | *Atelerix albiventris* (1, 0/0) | | |  |
| Mogom | 10°30'N/14°25'E | 29-31/01/2003 | 313 | | HD, N | | *Crocidura* sp. (8, 0/8) | | |  |
|  |  |  |  | |  | | *Mastomys natalensis* (3, 0/3) | | |  |
|  |  |  |  | |  | | *Arvicanthis* sp. (1, 0/1) | | |  |
|  |  |  |  | |  | | *Crocidura* sp. (2, 0/2) | | |  |
|  |  |  |  | |  | | *Arvicanthis* sp. (14, 0/12) | | |  |
|  |  |  |  | |  | | *Mastomys erythroleucus* (12, 0/12) | | |  |
|  |  |  |  | |  | | *M. kollmannspergeri* (15, 0/15) | | |  |
|  |  |  | Manual catches | | N | | *Desmodilliscus braueri* (2, 0/2) | | |  |
|  |  |  |  | |  | | *M. kollmannspergeri* (1, 0/1) | | |  |
| Diéra | 08°31'N/13°30'E | 11-12/02/2003 | 195 | | HD, N | | *Mastomys natalensis* (14, 0/12) | | |  |
|  |  |  |  | |  | | *Rattus rattus* (1, 0/1) | | |  |
|  |  |  |  | |  | | *Lemniscomys zebra* (7, 0/5) | | |  |
|  |  |  |  | |  | | *Arvicanthis* sp. (2, 0/2) | | |  |
|  |  |  |  | |  | | *M. kollmannspergeri* (2, 0/2) | | |  |
|  |  |  |  | |  | | *Taterillus congicus* (2, 0/2) | | |  |
| ***Total Cameroon*** |  |  | **1,109** | |  | | **Total specimens: 207, 0/182** | | |  |
|  | |  |  | |  | |  |  | |  |
| * Detailed data for Morocco available in ref. 60  ** No: number of specimens captured, I: number infected, T: number tested  F: Fields  N: Natural habitat  HD: Human dwellings  * F: Fields  N: Natura | | | | |  | |  |  | |  |
